# Crystal structure and characterization of a new chain-like polyrotaxane zinc(II) coordination polymer with mixed pyridine-2,6-di­carboxyl­ate and 1,4-bis­(1*H*-imidazol-1-ylmeth­yl)benzene ligands

**DOI:** 10.1107/S2056989025004608

**Published:** 2025-05-30

**Authors:** Chanikarn Kummuang, Kittipong Chainok, Nanthawat Wannarit

**Affiliations:** a Department of Chemistry Faculty of Science and Technology Thammasat University, Pathum Thani 12121 Thailand; b Thammasat University Research Unit in Multifunctional Crystalline Materials and Applications (TU-MCMA) Faculty of Science and Technology Thammasat University Pathum Thani 12121 Thailand; Vienna University of Technology, Austria

**Keywords:** Zn^II^, chain structure, polyrotaxane, 1,4-bis­(imidazol-1-ylmeth­yl)benzene, pyridine-2,6-di­carboxyl­ate, crystal structure

## Abstract

A new chain-like polyrotaxane Zn^II^ coordination polymer, *catena*-poly[[[[(pyridine-2,6-di­carboxyl­ato-κ^3^*O*,*N*,*O*′)zinc(II)]-μ-1,4-bis­(1*H*-imidazol-1-ylmeth­yl)benzene-κ^2^*N*^1^:*N*^1′^] hemi{bis­[μ-1,4-bis­(1*H*-imidazol-1-ylmeth­yl)benzene-κ^2^*N*^1^:*N*^1′^]bis­[(pyridine-2,6-di­carboxyl­ato-κ^3^*O*,*N*,*O*′)zinc(II)]}] nona­hydrate], {[Zn(C_7_H_3_NO_4_)(C_14_H_14_N_4_)][Zn_2_(C_7_H_3_NO_4_)_2_(C_14_H_14_N_4_)_2_]_0.5_·9H_2_O}_*n*_ or {[Zn_2_(2,6-PDC)_2_(bix)_2_]·9H_2_O}_*n*_ [where 2,6-PDC = pyridine-2,6-di­carboxyl­ate, C_7_H_3_NO_4_, and bix = 1,4-bis­(1*H*-imidazol-1-ylmeth­yl)benzene, C_14_H_14_N_4_], was synthesized and characterized. The mol­ecular structure comprises two Zn^II^ units. Both Zn^II^ ions are fivefold coordinated, with the coordination sphere inter­mediate between trigonal-bipyramidal and square-pyramidal, as defined by two bix ligands and one 2,6-PDC tridentate chelating ligand. For one of the units, two Zn^II^ ions are bridged by *gauche*-bix ligands, providing a dinuclear [Zn_2_(2,6-PDC)_2_(bix)_2_] macrocycle. The other unit is built by *trans*-bix bridging ligands, leading to a zigzag chain-like structure. Inter­estingly, these two entities are inter­weaved, leading to a polyrotaxane structure extending parallel to [101]. The crystal structure is consolidated by hydrogen-bonding, π–π and C—H⋯π inter­molecular inter­actions.

## Abstract 2

The crystal structure of a new polyrotaxane Zn^II^ coordination polymer, {[Zn_2_(2,6-PDC)_2_(bix)_2_]·9H_2_O}_*n*_, is comprised of dinuclear macrocyclic units and a zigzag chain-like structure extending parallel to [101].

## 1. Chemical context

Coordination polymers (CPs) are inorganic–organic materials composed of metal ions linked by organic ligands through coordinate-covalent bonds into structural units with different periodicity (Robin & Fromm, 2006 ▸; Batten *et al.*, 2012 ▸). These materials possess potential for applications in various fields, such as gas storage, separation, luminescence, magnetism, catalysis, and drug delivery (Fromm *et al.*, 2009 ▸; Batten *et al.*, 2016 ▸; Kothawade & Shende, 2024 ▸; Dragutan *et al.*, 2024 ▸). Among these materials, CPs of Zn^II^ are very attractive due to their varieties of structural arrangements and also their properties. The Zn^II^ atom has an [Ar]3*d*^10^ closed-shell electron configuration, and corresponding structure–property relationships are studied for applications like luminescence (Parmar *et al.*, 2020 ▸; Diana *et al.*, 2021 ▸; Li *et al.*, 2023 ▸). To create novel Zn^II^ CPs with inter­esting structures and properties, mixed *O*- and *N*-donor ligands can be utilized (Robin *et al.*, 2006 ▸; Du *et al.*, 2013 ▸). Bridging *O*-donor ligands, in particular heterocyclic aromatic di­carb­oxy­lic acid ligands such as pyridinedi­carboxyl­ate (PDC), can provide a variety of coordination modes with central metal ions, yielding a variety of framework periodicities and topologies (Gao *et al.*, 2006 ▸). Furthermore, the selection of ligands with carboxyl­ate groups and aromatic rings can promote hydrogen-bonding and π–π inter­molecular inter­actions, respectively, thus contributing to the stabilization of the crystal structure. For *N*-donor bridging ligands, the incorporation of flexible di­imidazole ligands such as 1,4-bis­(imidazol-1-ylmeth­yl)benzene (bix), which consists of two imidazole rings linked by a methyl­ene group to a benzene ring, can result in two possible coordination conformations with the central metal ion, *gauche* and *trans*, and consequently leads to a variety of extended CP periodicities and topologies (Tripuramallu *et al.*, 2012 ▸; Adarsh *et al.*, 2016 ▸; Li *et al.*, 2018 ▸). Zn^II^ CPs containing mixed PDC and *trans*-bix derivatives have been reported, for instance in the form of a grid structure [Zn(2,3-PDCO)(bix)·H_2_O]_*n*_ (2,3-PDCOH_2_ = pyridine-2,3-di­carb­oxy­lic acid *N*-oxide) (Wen *et al.*, 2009 ▸), a corrugated network [Zn(2,6-PDC)(bmix)_0.5_]_*n*_ [2,6-PDCH_2_ = pyridine-2,6-di­carb­oxy­lic acid and bmix = 1,4-bis­(2-methyl­imidazole-1-ylmeth­yl)benzene; Liu *et al.*, 2011 ▸], and a zigzag chain [Zn(3,4-PDC)(bix)]_*n*_ (3,4-PDCH_2_ = pyridine-3,4-di­carb­oxy­lic acid) (Voda *et al.*, 2017 ▸). Notably, the flexibility of the bix ligand with both *gauche-* and *trans-*conformations can facilitate the formation of inter­esting Zn^II^ CP topologies, for example a mono-periodic polyrotaxane, {[Zn(Or)(bix)(H_2_O)]_2_·6H_2_O}_*n*_ (OrK = potassium orotate) (Somnath *et al.*, 2022 ▸).
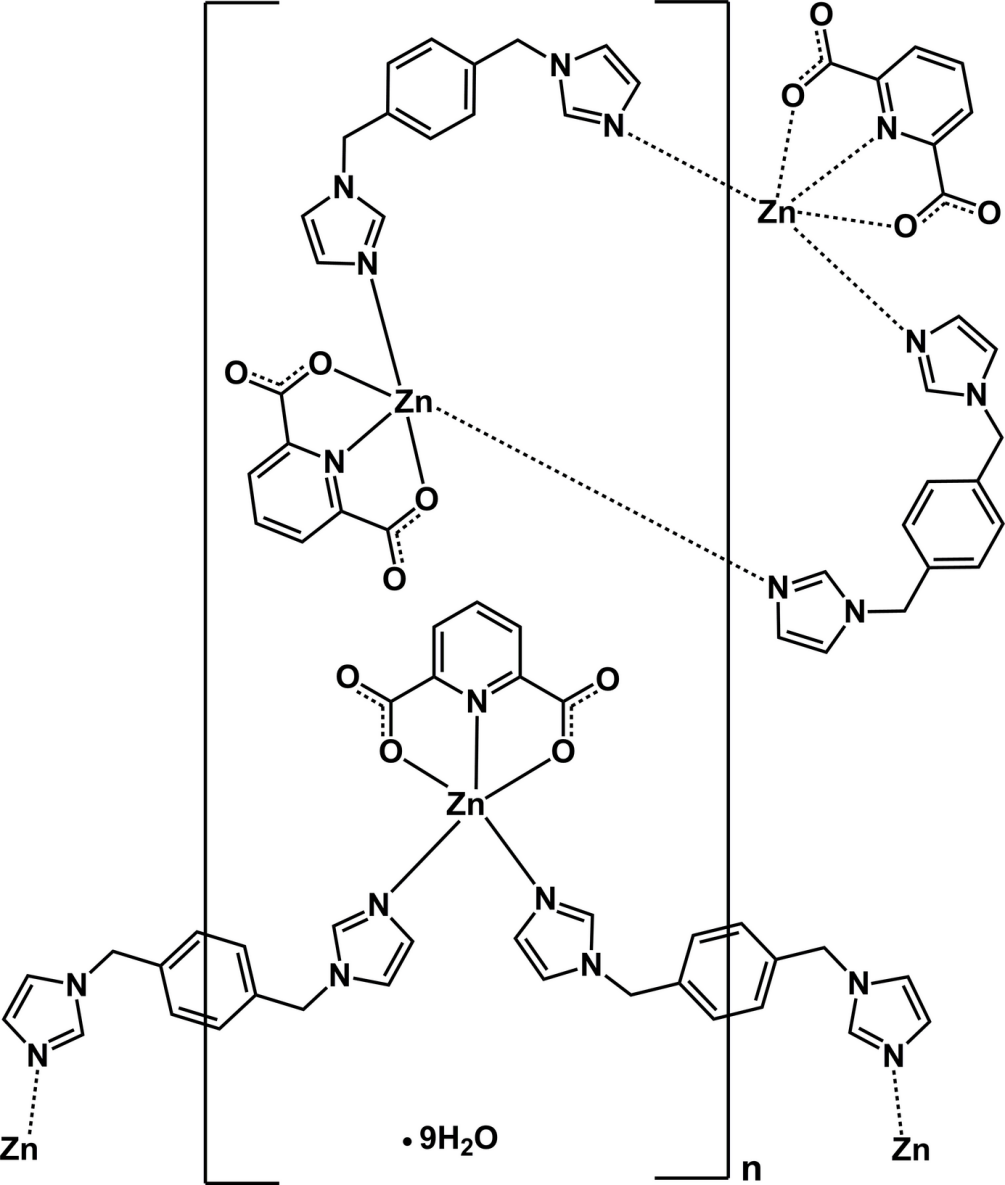


The current report is part of a study aimed at the synthesis of new Zn^II^ CPs using pyridine-2,6-di­carboxyl­ate (2,6-PDC) and 1,4-bis­(imidazol-1-ylmeth­yl)benzene (bix) by investigation of various synthetic conditions, including compositions, solvents, and methods, leading to a new chain-like polyrotaxane Zn^II^ CP, {[Zn_2_(2,6-PDC)_2_(bix)_2_]·9H_2_O}_*n*_.

## 2. Structural commentary

**Figure 1:**
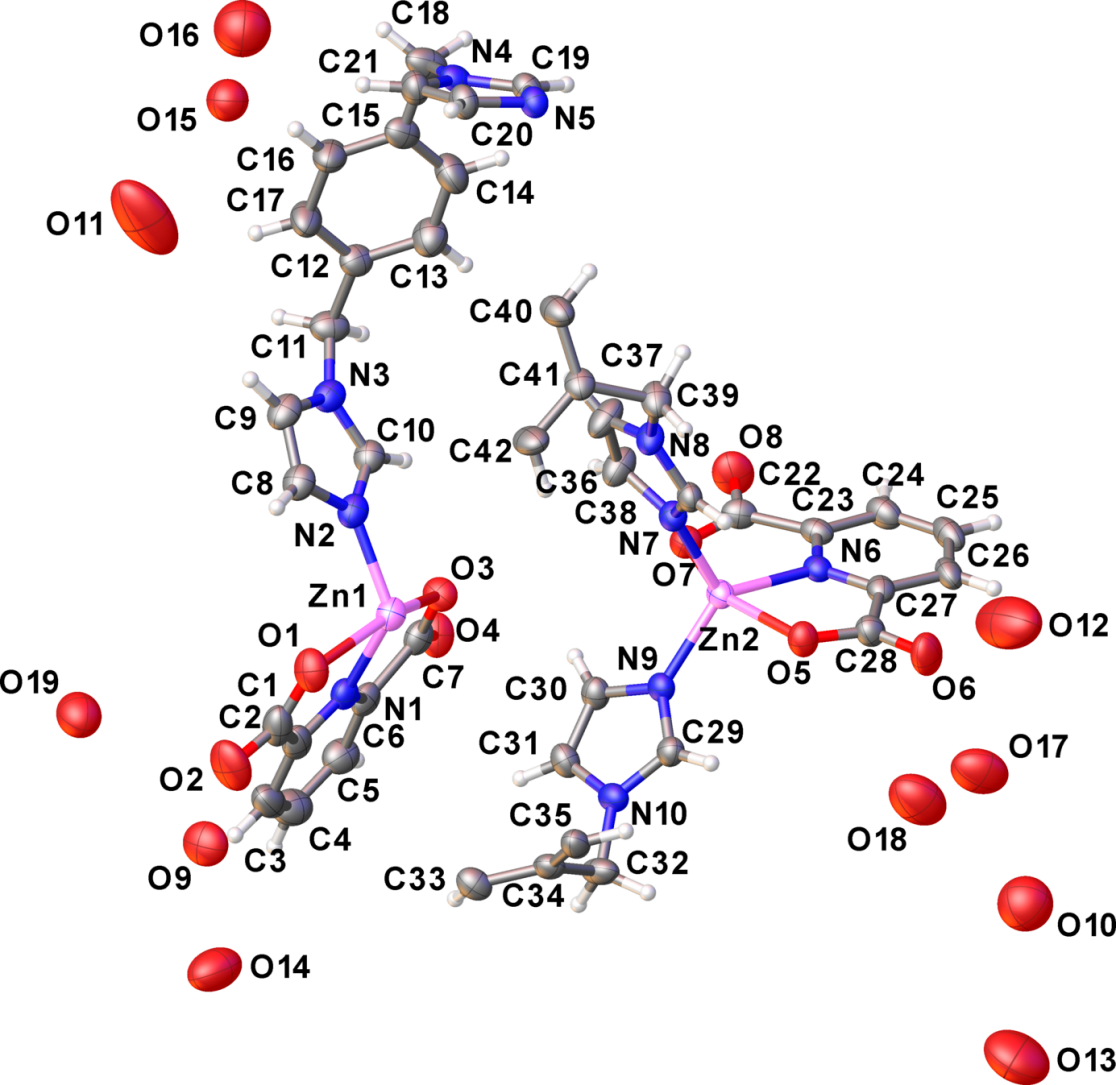
The asymmetric unit of the title compound. Zn1 is located in the center of inversion symmetry positions and two half-bix ligands sit across a twofold rotation axis. Displacement ellipsoids are drawn at the 30% probability level.

**Figure 2:**
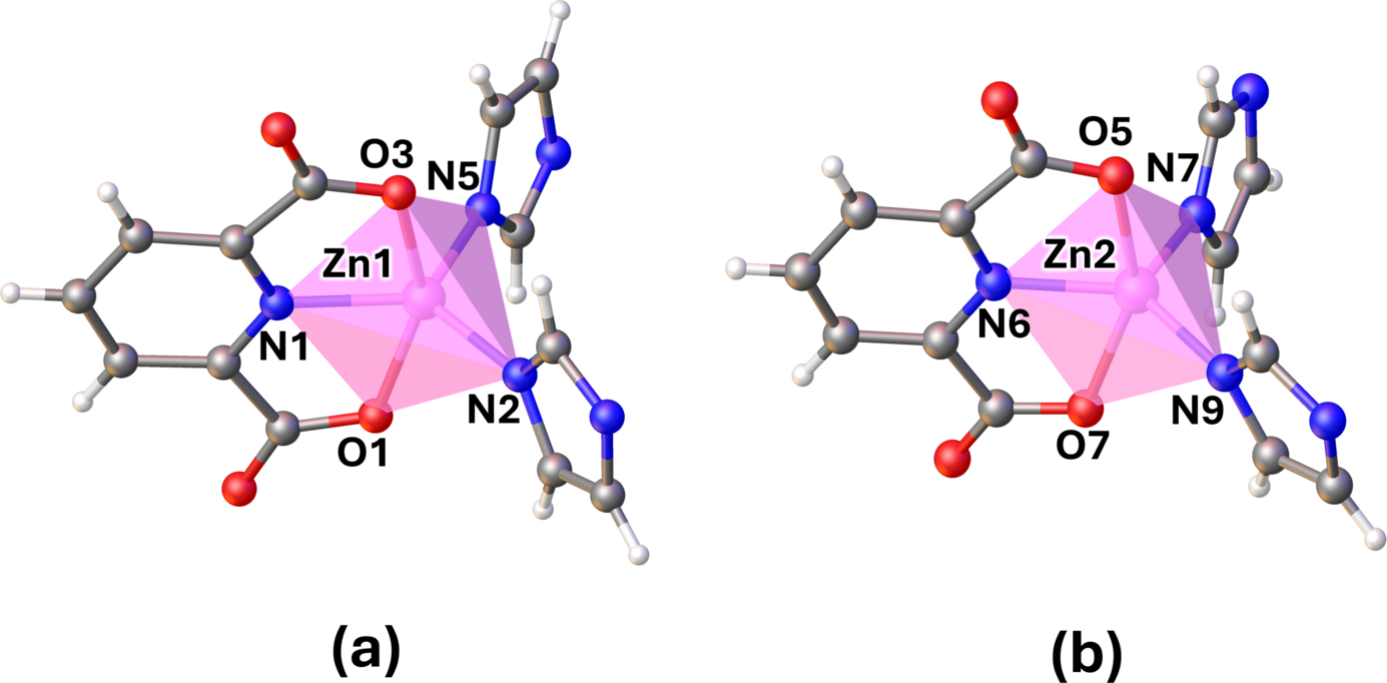
The coordination environment of the central Zn^II^ atoms in the title compound.

**Figure 3:**
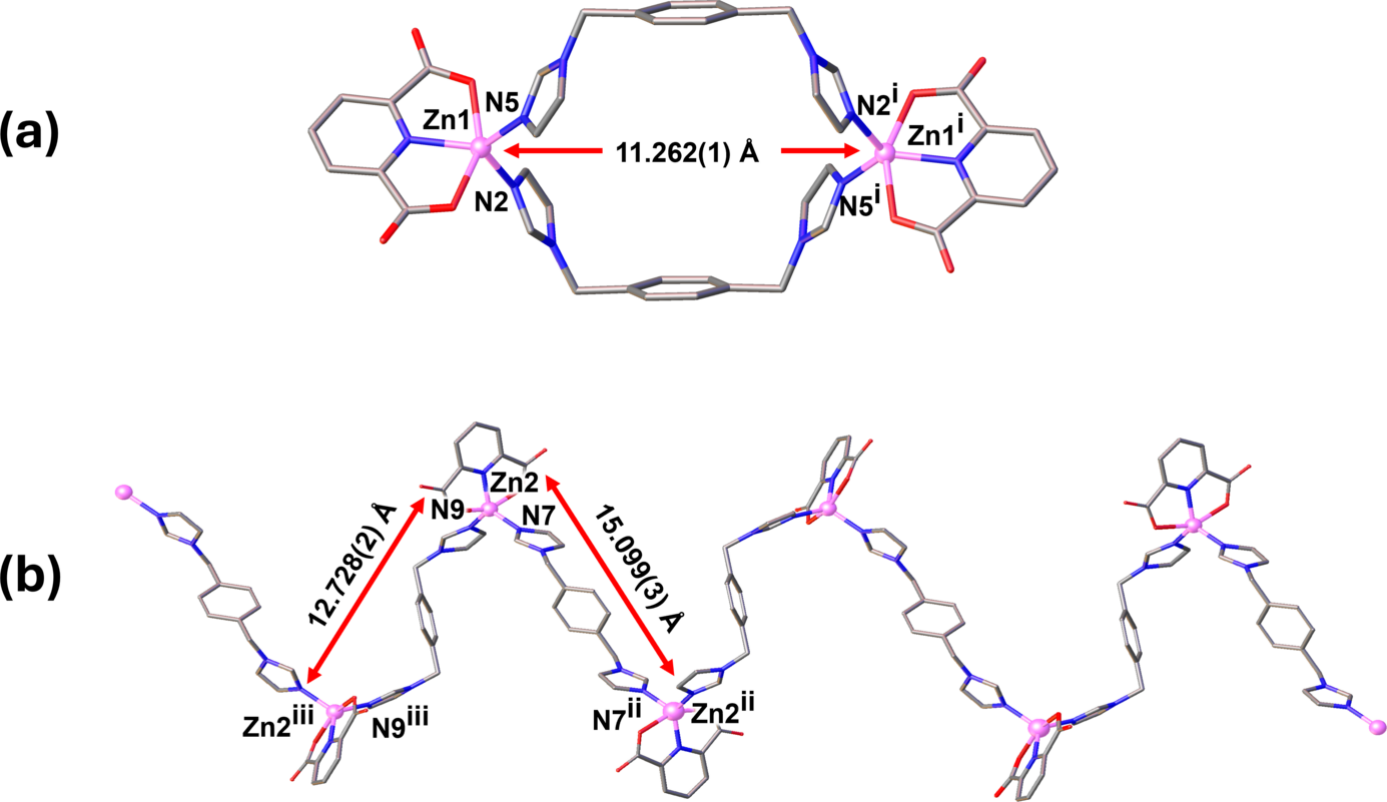
Two independent units: (*a*) the dinuclear Zn1 macrocyclic unit and (*b*) the zigzag coordination polymeric chain-like structure of the Zn2 unit of the title compound.

**Figure 4:**
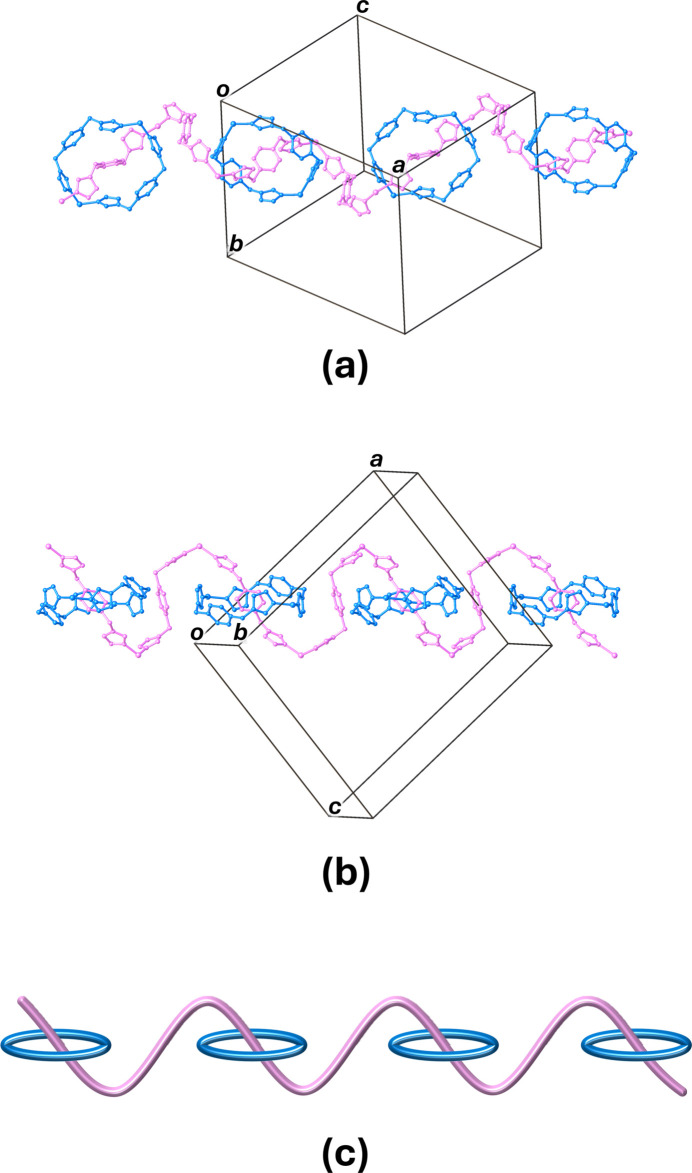
(*a*) Top, (*b*) side views and (*c*) schematic representation of the mono-periodic polyrotaxane structure of the title compound. The 2,6-DPC, hydrogen atoms and non-coordinating water mol­ecules are omitted for clarity.

The asymmetric unit of the title compound consists of two Zn^II^ atoms, one bix and two half-bix ligands, which sit across a twofold rotation axis, two 2,6-PDC ligands and nine non-coordinating water mol­ecules (Fig. 1 ▸). Selected bond lengths and angles are listed in Table 1 ▸. The coordination number of both the Zn1 and Zn2 atoms is 5 (Fig. 2 ▸). The environment of Zn1 is defined by two *N*-donor atoms from two bix ligands and one N and two O atoms from a terminal 2,6-PDC tridentate chelating ligand. The Zn1—N distances range from 1.984 (3) to 2.014 (3) Å, and the Zn1—O distances are 2.151 (3) and 2.208 (3) Å. The structural parameter τ_5_ of Zn1 is 0.46 (Fig. S1 in the electronic supporting information, ESI), and thus the coordination environment is inter­mediate between a square pyramid and a trigonal bipyramid (τ = 0 for an ideal square pyramid and τ = 1 for an ideal trigonal bipyramid; Addison *et al.*, 1984 ▸). Two Zn1 ions are linked by two *gauche-*bix bridging ligands with μ-κ^2^*N*:*N′*-coordination mode, forming a dinuclear [Zn_2_(2,6-PDC)_2_(bix)_2_] macrocyclic unit (Fig. 3 ▸*a*). The Zn1⋯Zn1^i^ distance across the dinuclear unit is 11.262 (1) Å [symmetry code: (i) −*x* + 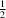, −*y* − 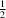, −*z* + 1). The environment around Zn2 is defined by two *N*-donor atoms from two bix ligands and one *N*- and two *O*-donor atoms from a terminal 2,6-PDC tridentate chelating ligand. The Zn2—N distances are in the range 1.984 (3) to 2.010 (3) Å and the Zn2—O distances are 2.172 (3) and 2.209 (3) Å. The value of τ_5_ is 0.42 (Fig. S1 in the ESI), indicating an inter­mediate five-coordinate environment similar to the degree of distortion found around Zn1. The Zn2 atoms are linked by the *trans-*bix bridging ligand with μ-κ^2^*N*:*N′*-coordination modes, leading to a zigzag chain-like structure extending parallel to [101] (Fig. 3 ▸*b* and Fig. S1 in the ESI). The Zn2⋯Zn2^ii^ and Zn2⋯Zn2^iii^ distances are 15.099 (3) and 12.728 (2) Å, respectively [symmetry codes: (ii) −*x* + 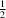, −*y* + 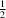, −*z* + 2; (iii) = −*x*, *y*, −*z* + 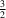]. Inter­estingly, the zigzag chains involving Zn2 represent the threading into the dinuclear [Zn_2_(2,6-PDC)_2_(bix)_2_] macrocyclic units, providing an extended mono-periodic polyrotaxane structure parallel to [101], as shown in Fig. 4 ▸ and Fig. S2 in the ESI.

**Figure 5:**
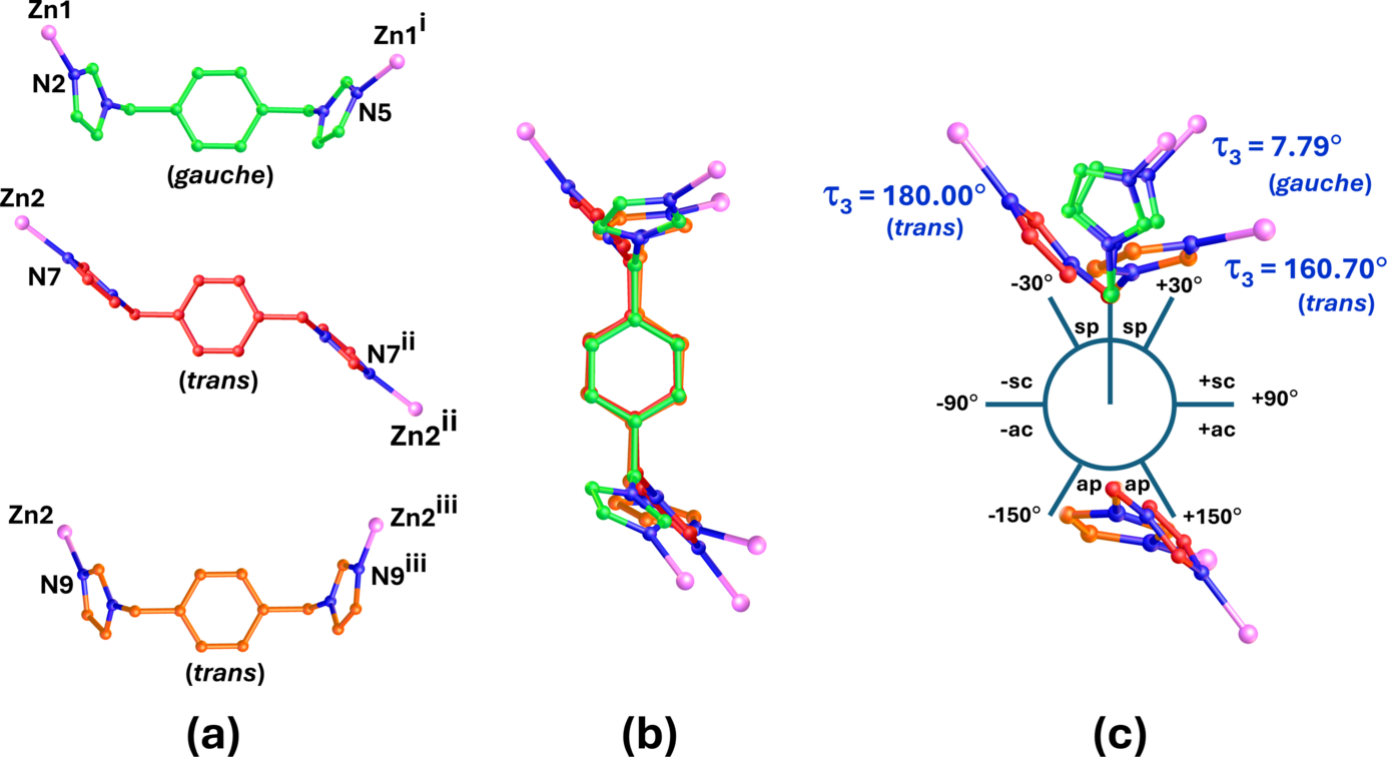
Views of (*a*) conformations, (*b*) overlay and (*c*) Newmann projection representation of three different bix ligands in the title compound. [Symmetry codes: (i) −*x* + 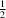, −*y* − 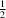, −*z* + 1; (ii) −*x* + 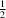, −*y* + 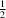, −*z* + 2; (iii) = −*x*, *y*, −*z* + 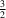.]

The bix ligands, crucial for the polyrotaxane structure of the title compound, exhibit three distinct conformations, as shown in Fig. 5 ▸ and Figs. S2–S4 in the ESI. Geometrical parameters characterizing these conformations are summarized in Table S1 in the ESI. In the bix ligand within the Zn1 unit, the imidazole rings {N2N3;N4N5} display a synperiplanar conformation, characterized by a torsion angle (τ_3_) of 7.90° (through N3—C11—C18—N4). The imidazole rings are twisted with respect to the phenyl ring with torsion angles (τ_1_) of 67.4 (6)° (through C9—N3—C11—C12) and (τ_2_) of −83.8 (5)° (through C21—N4—C18—C15), indicating a *gauche* conformation. The two independent bix ligands in the Zn2 unit, related by twofold rotation symmetry, show different conformations. For the {N7N8;N8′N7′} rings, an anti­periplanar twist is observed, with a torsion angle (τ_3_) of 180.00° (through N8—C39—C39′—N8′). The twist relative to the phenyl ring is defined by torsion angles (τ_1_) and (τ_2_) of −48.90 and 48.90°, respectively. For the {N9N10;N10′N9′} rings, the imidazole rings show a near anti­periplanar conformation, with a torsion angle (τ_3_) of 160.10° (through N10—C32—C32′—N10′), and the twist relative to the phenyl ring defined by a torsion angles (τ_1_ and τ_2_) of 78.7 (5)°. These parameters correspond to a *trans*-conformation for the Zn2 unit. Consequently, the difference of these bix conformations also affect the metal–metal separation (Tripuramallu *et al.*, 2012 ▸) in the title compound.

## 3. Supra­molecular features

**Figure 6:**
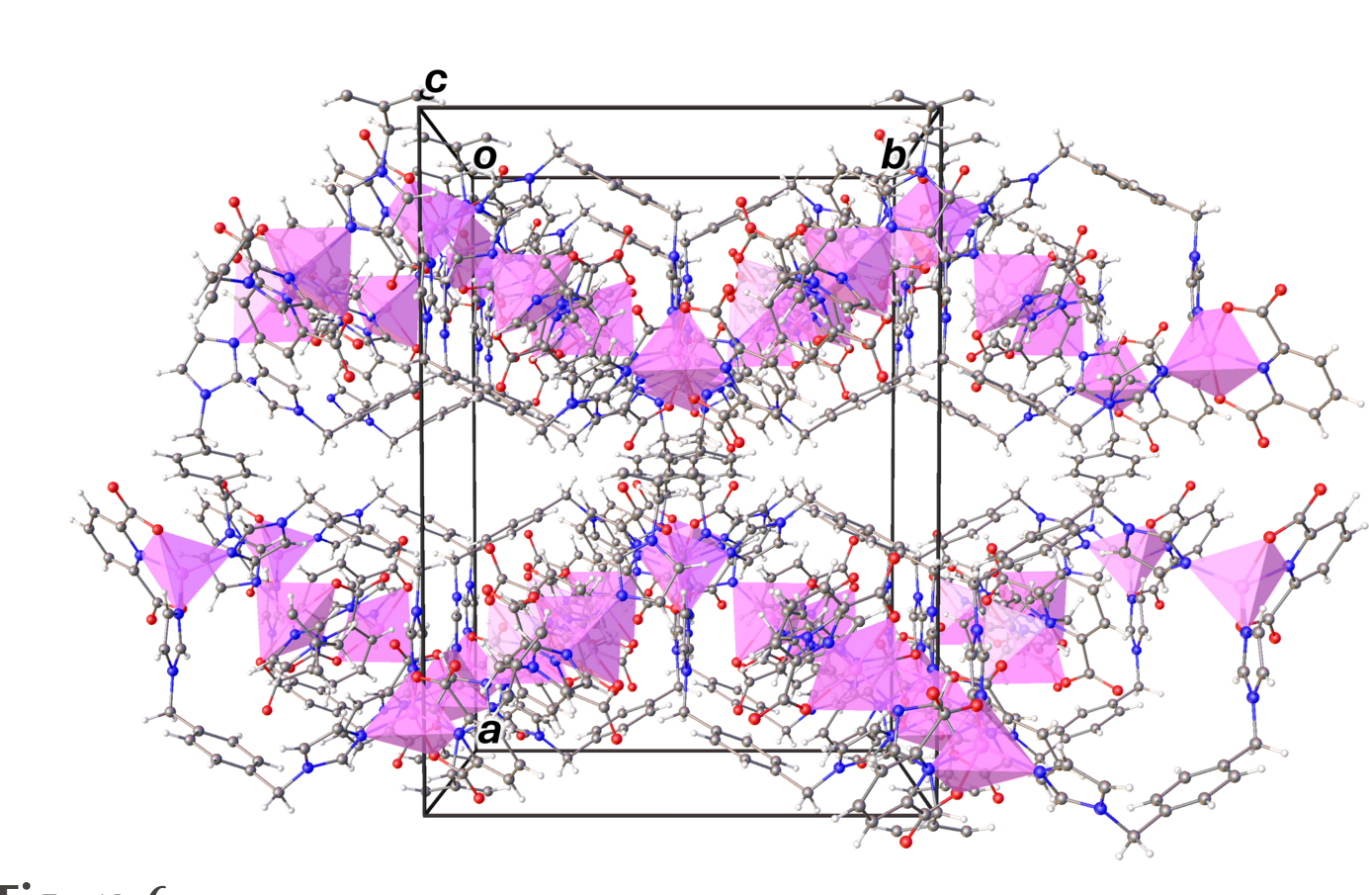
The crystal packing of the title compound. The water mol­ecules are omitted for clarity.

The crystal structure of the title compound is consolidated by the presence of a variety of inter­molecular inter­actions, namely hydrogen-bonding, π–π and C —H⋯π inter­actions, as detailed in Tables 2 ▸ and 3 ▸. It is noted that the hydrogen atoms bonded to the oxygen atoms of non-coordinating water mol­ecules were not assigned in the structure, but the O⋯O separations [O11⋯O12 = 3.096 (14) Å, O12⋯O13 = 3.214 (16) Å, O13⋯O14 = 2.882 (9) Å, O10⋯O17 = 3.120 (16) Å, O17⋯O18 = 2.862 (14) Å, O16⋯O18 = 3.206 (19) Å, O9⋯O18 = 2.876 (9) Å] suggest the presence of medium-to-weak hydrogen-bonding inter­actions between them. Non-classical inter­molecular hydrogen-bonding C—H⋯O inter­actions are found between (i) the C—H group of the imidazole ring on the bix ligands and the oxygen atoms of the carboxyl­ate group of the 2,6-PDC ligands (Fig. S5 in the ESI), and (ii) between the C—H groups of both the imidazole ring and the –CH_2_ group on the bix ligands and the non-coordinating water mol­ecules (Fig. S6 in the ESI). The π–π inter­actions are observed between the pyridine rings of 2,6-PDC ligands in adjacent zigzag chains with a centroid-to-centroid distance *Cg*5⋯*Cg*5^i^ of 3.646 (2) Å and a slippage of 1.434 Å [*Cg*5 is the centroid of the N6/C23–C27 ring; symmetry code: (i) −*x* + 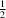, −*y* + 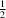, −*z* + 1]. Additionally, π–π inter­actions are found between the pyridine rings of 2,6-PDC ligands in the adjacent zigzag chains and the dinuclear units with a centroid-to-centroid distance *Cg*5⋯*Cg*12^i^ of 3.729 (3) Å and a slippage of 0.782 and 0.686 Å (*Cg*5 and *Cg*12 are the centroids of the N6/C23–C27 and N1/C2–C6 rings), as shown in Fig. S7 in the ESI. Furthermore, C—H⋯π inter­actions are observed between the C—H groups of 2,6-PDC in the dinuclear units and the imidazole ring of the bix ligand in the zigzag chains, C5—H5⋯*Cg*3^v^ and C26—H26⋯*Cg*10^v^ [symmetry code: (v) −*x* + 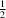, −*y* + 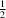, −*z* + 1]. Additional inter­actions occur between a C—H group of the benzene ring of a bix ligand in the zigzag chains and the imidazole ring of the bix ligand in the dinuclear units, namely C42— H42⋯*Cg*11^iv^ [where *Cg*3, *Cg*10 and *Cg*11 are the centroids of the five-membered N7/C36–C38, N2/C8–C10 and N4/C19–C21 rings, respectively; symmetry code: (iv) *x*, −*y*, *z* + 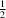], as shown in Fig. S8 in the ESI. The crystal packing of the title compound is shown in Fig. 6 ▸ and Fig. S9 in the ESI.

## 4. Spectroscopic, powder X-ray diffraction (PXRD) and thermal properties

The FT-IR spectrum of the title compound (Fig. S10 in the ESI) shows a strong broad band centered at 3434 cm^−1^, assigned to the O—H stretching of water mol­ecules. A band at 3127 cm^−1^ is assigned to the C—H stretching of aromatic rings for both ligands. The characteristic bands found at 1639 and 1421 cm^−1^ can be assigned to be the asymmetric and symmetric stretching vibrations of the carboxyl­ate group of the 2,6-PDC ligand. Bands appearing at 1533 and 1097 cm^−1^ could be assigned to the C=N and C—N stretching, respectively, and the bands in the range of 700–500 cm^−1^ to C—H bending vibration of bix ligands (Tripuramallu *et al.*, 2012 ▸; Somnath *et al.*, 2022 ▸).

The PXRD patterns of the title compound are shown in Fig. S11 in the ESI, revealing a good match between the calculated pattern from single-crystal data and the experimental data of the as-synthesized compound, indicating that it was synthesized in a phase-pure manner.

To study the thermal stability of the title compound, thermogravimetric analysis (TGA) was performed in the temperature range of 303–1073 K under nitro­gen atmosphere. From the TGA curve shown in Fig. S12 in the ESI, the first degradation step in the range of 303–403 K represents a mass loss of 17.30%, corresponding to the loss of the nine water mol­ecules (calculated 17.45%). Then the resulting compound remained stable up to 573 K. The degradation steps observed in the temperature range of 573–833 K represents a mass loss of 51.59%, indicating the release of two bix and two 2,6-PDC ligands (calculated 50.74%). The remaining residue at high temperature could be assigned to ZnO.

## 5. Database survey

A search of the Cambridge Structural Database (CSD, version 5.44, last update April 2023; Groom *et al.*, 2016 ▸) using the ConQuest software (Bruno *et al.*, 2002 ▸) for structures of mixed 2,6-PDC and bix ligand-based Zn^II^ polyrotaxane CPs yielded no hits. To the best of our knowledge, only one relevant Zn^II^ CP containing mixed di­carboxyl­ate, orotate (Or), and bix ligands, {[Zn(Or)(bix)(H_2_O)]_2_·6H_2_O}_*n*_ (PEFHAN; Somnath *et al.*, 2022 ▸), exhibiting a chain-like polyrotaxane structure, has been reported. Although the crystal structure of this compound exhibits both *gauche* and *trans* conformations of the bix ligand (like in the title compound), differences in moieties such as the orotate ligand and the number of water mol­ecules, as well as variations in the degree of bix mol­ecule flexibility and supra­molecular inter­actions, make a direct comparison difficult.

## 6. Synthesis and crystallization

All chemicals purchased were reagent-grade and used without further purification. The bix ligand was prepared according to a literature procedure (Hoskins *et al.*, 1997 ▸). A dimethyl formamide solution (10 ml) of bix·2H_2_O (0.2383 g, 1 mmol) was added to an aqueous solution (10 ml) of Zn(NO_3_)_2_·6H_2_O (0.2975 g, 1 mmol) and stirred for 10 min at 333 K. Subsequently, a mixture of an aqueous solution (10 ml) of 2,6-PDCH_2_ (0.1671 g, 1 mmol) and NaOH (0.0845 g, 2 mmol) was added, and the resulting mixture was stirred for 20 min, giving a colorless precipitate. Then, a mixed solution of DMF (10 ml) and deionized water (10 ml) was slowly added, and the mixture was stirred for 40 min. The solution became clear and colorless. This solution was filtered and allowed to slowly evaporate at room temperature. Colorless, block-shaped crystals of the title compound were obtained within one week (17.6% yield, based on the Zn^II^ salt).

## 7. Refinement

Crystal data, data collection and structure refinement details are summarized in Table 4 ▸. Hydrogen atoms bonded to carbon atoms were placed at calculation positions and refined isotropically using a riding model with C—H = 0.93 Å and *U*_iso_(H) = 1.2*U*_eq_(C) for aromatic hydrogen atoms, and C—H = 0.97 Å, *U*_iso_(H) = 1.2*U*_eq_(C) for methyl­ene hydrogen atoms. The hydrogen atoms bonded to the oxygen atoms of the non-coordinating water mol­ecules (O9–O19) could not be located reliably and thus were not included in the model, but were taken into account in the overall formula. Some of non-coordinating water mol­ecules were refined with site occupancies of 0.75 for O10 and O12 and 0.5 for O17, while other water mol­ecules (O15 and O16) were found to be disordered with site occupancies of 0.5.

## Supplementary Material

Crystal structure: contains datablock(s) I. DOI: 10.1107/S2056989025004608/wm5758sup1.cif

Supporting Information. DOI: 10.1107/S2056989025004608/wm5758sup4.pdf

CCDC reference: 2453300

Additional supporting information:  crystallographic information; 3D view; checkCIF report

## Figures and Tables

**Table 1:** Selected geometric parameters (Å, °)

Zn1—O1	2.208 (3)	Zn2—O5	2.172 (3)
Zn1—O3	2.151 (3)	Zn2—O7	2.209 (3)
Zn1—N1	2.014 (3)	Zn2—N6	2.010 (3)
Zn1—N2	1.984 (3)	Zn2—N7	1.984 (3)
Zn1—N5^i^	1.990 (3)	Zn2—N9	1.992 (3)
			
O3—Zn1—O1	152.50 (13)	O5—Zn2—O7	153.06 (10)
N1—Zn1—O1	75.70 (13)	N6—Zn2—O5	76.93 (11)
N1—Zn1—O3	77.05 (11)	N6—Zn2—O7	76.35 (11)
N2—Zn1—O1	98.27 (14)	N7—Zn2—O5	101.49 (12)
N2—Zn1—O3	94.35 (13)	N7—Zn2—O7	93.77 (11)
N2—Zn1—N1	125.19 (12)	N7—Zn2—N6	117.44 (11)
N2—Zn1—N5^i^	114.81 (12)	N7—Zn2—N9	114.82 (12)
N5^i^—Zn1—O1	97.97 (12)	N9—Zn2—O5	96.62 (12)
N5^i^—Zn1—O3	98.73 (11)	N9—Zn2—O7	96.92 (11)
N5^i^—Zn1—N1	119.99 (12)	N9—Zn2—N6	127.60 (11)

Symmetry code: (i) 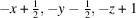.

**Table 2:** Hydrogen-bond geometry (Å, °) *Cg*3, *Cg*10 and *Cg*11 are the centroids of the N7/C36/C37/N8/C38/N2/C8/C9/N3/C10 and N4/C20/C21/N5/C19 rings, respectively.

*D*—H⋯*A*	*D*—H	H⋯*A*	*D*⋯*A*	*D*—H⋯*A*
C18—H18*A*⋯O19^ii^	0.97	2.45	3.421 (7)	174
C19—H19⋯O9^i^	0.93	2.44	3.328 (5)	160
C21—H21⋯O15^iii^	0.93	2.50	3.394 (10)	162
C21—H21⋯O16^iii^	0.93	2.41	3.278 (18)	154
C29—H29⋯O19	0.93	2.47	3.213 (6)	137
C30—H30⋯O4^iv^	0.93	2.53	3.149 (5)	124
C38—H38⋯O4^v^	0.93	2.58	2.988 (5)	107
C5—H5⋯*Cg*3^v^	0.93	2.81	3.624 (5)	146
C26—H26⋯*Cg*10^v^	0.93	2.61	3.443 (4)	150
C42—H42⋯*Cg*11^iv^	0.93	2.93	3.818 (5)	161

Symmetry codes: (i) 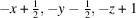; (ii) 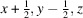; (iii) 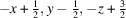; (iv) 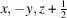; (v) 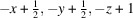.

**Table 3:** Analysis of short ring inter­actions (Å) *Cg*(I) and *Cg*(*J*) are the centroids of rings *I* and *J*; *CgI*_Perp is the perpendicular distance of *Cg*(*I*) on ring *J*, and slippage is the distance between *Cg*(*I*) and the perpendicular projection of *Cg*(*J*) on ring *I. *Cg**5, and *Cg*12 are the centroids of the N6/C23–C27 and N1/C2–C6 rings, respectively.

*Cg*(*I*)	*Cg*(*J*)	Symmetry_*Cg*(*J*)	*Cg*(*I*)⋯*Cg*(*J*)	*CgI*_Perp	*CgJ*_Perp	Slippage
*Cg*5	*Cg*5	-*x* + 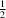 , −*y* + 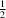 , −*z* + 1	3.646 (2)	3.3521 (15)	3.3521 (15)	1.434
*Cg*5	*Cg*12	-*x* + 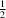 , −*y* + 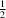 , −*z* + 1	3.729 (3)	3.6648 (15)	3.6455 (19)	0.782
*Cg*12	*Cg*5	-*x* + 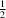 , −*y* + 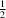 , −*z* + 1	3.728 (3)	3.6453 (19)	3.6648 (15)	0.686

**Table 4:** Experimental details

Crystal data	
Chemical formula	[Zn(C_7_H_3_NO_4_)(C_14_H_14_N_4_)][Zn_2_(C_7_H_3_NO_4_)_2_(C_14_H_14_N_4_)_2_]_0.5_·9H_2_O
*M*_r_	1099.70
Crystal system, space group	Monoclinic, *C*2/*c*
Temperature (K)	296
*a*, *b*, *c* (Å)	25.577 (4), 18.632 (3), 22.463 (4)
β (°)	94.335 (6)
*V* (Å^3^)	10674 (3)
*Z*	8
Radiation type	Mo *K*α
μ (mm^−1^)	0.97
Crystal size (mm)	0.24 × 0.2 × 0.2

Data collection	
Diffractometer	Bruker D8 QUEST CMOS PHOTON II
Absorption correction	Multi-scan (*SADABS*; Krause *et al.*, 2015 ▸)
*T*_min_, *T*_max_	0.685, 0.746
No. of measured, independent and observed [*I* > 2σ(*I*)] reflections	129120, 9915, 8159
*R*_int_	0.058
(sin θ/λ)_max_ (Å^−1^)	0.606

Refinement	
*R*[*F*^2^ > 2σ(*F*^2^)], *wR*(*F*^2^), *S*	0.055, 0.185, 1.05
No. of reflections	9915
No. of parameters	658
No. of restraints	18
H-atom treatment	H-atom parameters constrained
Δρ_max_, Δρ_min_ (e Å^−3^)	1.20, −0.56

Computer programs: *APEX3* and *SAINT* (Bruker, 2016 ▸), *OLEX2.solve* (Bourhis *et al.*, 2015 ▸), *SHELXL* (Sheldrick, 2015 ▸), *OLEX2* (Dolomanov *et al.*, 2009 ▸) and *publCIF* (Westrip, 2010 ▸).

## supplementary crystallographic information

### catena-Poly[[[[(pyridine-2,6-dicarboxylato-κ3O,N,O')zinc(II)]-µ-1,4-bis(1H-imidazol-1-ylmethyl)benzene-κ2N1:N1'] hemi{bis[µ-1,4-bis(1H-imidazol-1-ylmethyl)benzene-κ2N1:N1']bis[(pyridine-2,6-dicarboxylato-κ3O,N,O')zinc(II)]}] nonahydrate] Crystal data

**Table:**

[Zn(C_7_H_3_NO_4_)(C_14_H_14_N_4_)][Zn_2_(C_7_H_3_NO_4_)_2_(C_14_H_14_N_4_)_2_]_0.5_·9H_2_O	*F*(000) = 4416
*M**_r_* = 1099.70	*D*_x_ = 1.369 Mg m^−^^3^
Monoclinic, *C*2/*c*	Mo *K*α radiation, λ = 0.71073 Å
*a* = 25.577 (4) Å	Cell parameters from 9980 reflections
*b* = 18.632 (3) Å	θ = 2.6–25.9°
*c* = 22.463 (4) Å	µ = 0.97 mm^−^^1^
β = 94.335 (6)°	*T* = 296 K
*V* = 10674 (3) Å^3^	Block, clear colourless
*Z* = 8	0.24 × 0.2 × 0.2 mm

### catena-Poly[[[[(pyridine-2,6-dicarboxylato-κ3O,N,O')zinc(II)]-µ-1,4-bis(1H-imidazol-1-ylmethyl)benzene-κ2N1:N1'] hemi{bis[µ-1,4-bis(1H-imidazol-1-ylmethyl)benzene-κ2N1:N1']bis[(pyridine-2,6-dicarboxylato-κ3O,N,O')zinc(II)]}] nonahydrate] Data collection

**Table:**

Bruker D8 QUEST CMOS PHOTON II diffractometer	9915 independent reflections
Radiation source: sealed x-ray tube, Mo	8159 reflections with *I* > 2σ(*I*)
Graphite monochromator	*R*_int_ = 0.058
Detector resolution: 7.39 pixels mm^-1^	θ_max_ = 25.5°, θ_min_ = 2.6°
ω and φ scans	*h* = −30→30
Absorption correction: multi-scan (SADABS; Krause *et al.*, 2015)	*k* = −22→22
*T*_min_ = 0.685, *T*_max_ = 0.746	*l* = −27→27
129120 measured reflections	

### catena-Poly[[[[(pyridine-2,6-dicarboxylato-κ3O,N,O')zinc(II)]-µ-1,4-bis(1H-imidazol-1-ylmethyl)benzene-κ2N1:N1'] hemi{bis[µ-1,4-bis(1H-imidazol-1-ylmethyl)benzene-κ2N1:N1']bis[(pyridine-2,6-dicarboxylato-κ3O,N,O')zinc(II)]}] nonahydrate] Refinement

**Table:**

Refinement on *F*^2^	Primary atom site location: iterative
Least-squares matrix: full	Hydrogen site location: inferred from neighbouring sites
*R*[*F*^2^ > 2σ(*F*^2^)] = 0.055	H-atom parameters constrained
*wR*(*F*^2^) = 0.185	*w* = 1/[σ^2^(*F*_o_^2^) + (0.1223*P*)^2^ + 11.6285*P*] where *P* = (*F*_o_^2^ + 2*F*_c_^2^)/3
*S* = 1.05	(Δ/σ)_max_ < 0.001
9915 reflections	Δρ_max_ = 1.20 e Å^−^^3^
658 parameters	Δρ_min_ = −0.56 e Å^−^^3^
18 restraints	

### catena-Poly[[[[(pyridine-2,6-dicarboxylato-κ3O,N,O')zinc(II)]-µ-1,4-bis(1H-imidazol-1-ylmethyl)benzene-κ2N1:N1'] hemi{bis[µ-1,4-bis(1H-imidazol-1-ylmethyl)benzene-κ2N1:N1']bis[(pyridine-2,6-dicarboxylato-κ3O,N,O')zinc(II)]}] nonahydrate] Special details

**Table:**

Geometry. All esds (except the esd in the dihedral angle between two l.s. planes) are estimated using the full covariance matrix. The cell esds are taken into account individually in the estimation of esds in distances, angles and torsion angles; correlations between esds in cell parameters are only used when they are defined by crystal symmetry. An approximate (isotropic) treatment of cell esds is used for estimating esds involving l.s. planes.

### catena-Poly[[[[(pyridine-2,6-dicarboxylato-κ3O,N,O')zinc(II)]-µ-1,4-bis(1H-imidazol-1-ylmethyl)benzene-κ2N1:N1'] hemi{bis[µ-1,4-bis(1H-imidazol-1-ylmethyl)benzene-κ2N1:N1']bis[(pyridine-2,6-dicarboxylato-κ3O,N,O')zinc(II)]}] nonahydrate] Fractional atomic coordinates and isotropic or equivalent isotropic displacement parameters (Å^2^)

**Table:**

	*x*	*y*	*z*	*U*_iso_*/*U*_eq_	Occ. (<1)
Zn1	0.15753 (2)	−0.00657 (2)	0.38691 (2)	0.05507 (16)	
O1	0.09238 (14)	0.02720 (19)	0.43969 (15)	0.0806 (9)	
O2	0.03170 (16)	0.1129 (2)	0.4428 (2)	0.1164 (15)	
O3	0.21169 (11)	0.01072 (15)	0.31956 (12)	0.0617 (7)	
O4	0.23136 (13)	0.08944 (16)	0.24967 (14)	0.0762 (8)	
N1	0.13180 (11)	0.08573 (16)	0.34825 (13)	0.0519 (7)	
N2	0.21307 (14)	−0.01413 (17)	0.45320 (14)	0.0575 (8)	
N3	0.29250 (14)	−0.01172 (17)	0.49653 (14)	0.0590 (8)	
N4	0.41613 (12)	−0.30010 (16)	0.64954 (15)	0.0534 (7)	
N5	0.37235 (12)	−0.40007 (15)	0.64217 (12)	0.0502 (7)	
C1	0.06868 (18)	0.0836 (3)	0.4211 (2)	0.0782 (13)	
C2	0.08967 (15)	0.1181 (2)	0.3671 (2)	0.0646 (10)	
C3	0.0693 (2)	0.1792 (3)	0.3373 (3)	0.0839 (15)	
H3	0.039789	0.202290	0.349763	0.101*	
C4	0.0940 (2)	0.2043 (3)	0.2890 (3)	0.0919 (16)	
H4	0.081031	0.244602	0.268468	0.110*	
C5	0.1378 (2)	0.1700 (2)	0.2710 (2)	0.0756 (12)	
H5	0.154709	0.186772	0.238462	0.091*	
C6	0.15605 (15)	0.11016 (19)	0.30231 (16)	0.0558 (9)	
C7	0.20415 (15)	0.0669 (2)	0.28917 (16)	0.0565 (9)	
C8	0.21003 (19)	−0.0091 (2)	0.51427 (18)	0.0644 (10)	
H8	0.179265	−0.007743	0.533826	0.077*	
C9	0.25897 (19)	−0.0067 (2)	0.54066 (18)	0.0662 (11)	
H9	0.268183	−0.002293	0.581348	0.079*	
C10	0.26350 (17)	−0.0158 (2)	0.44476 (17)	0.0581 (9)	
H10	0.277066	−0.019253	0.407606	0.070*	
C11	0.35050 (18)	−0.0073 (2)	0.5032 (2)	0.0710 (12)	
H11A	0.360660	0.036798	0.524015	0.085*	
H11B	0.363770	−0.005252	0.463943	0.085*	
C12	0.37511 (16)	−0.0696 (2)	0.53687 (18)	0.0604 (9)	
C13	0.3928 (2)	−0.1296 (3)	0.5080 (2)	0.0829 (13)	
H13	0.388063	−0.132804	0.466656	0.100*	
C14	0.4176 (2)	−0.1847 (3)	0.5403 (3)	0.0843 (14)	
H14	0.429743	−0.224224	0.520210	0.101*	
C15	0.42455 (16)	−0.1821 (2)	0.6010 (2)	0.0620 (10)	
C16	0.4063 (2)	−0.1235 (2)	0.6298 (2)	0.0773 (12)	
H16	0.410162	−0.121374	0.671218	0.093*	
C17	0.3820 (2)	−0.0670 (2)	0.5978 (2)	0.0796 (13)	
H17	0.370417	−0.027233	0.618107	0.096*	
C18	0.45194 (16)	−0.2418 (2)	0.6356 (2)	0.0744 (12)	
H18A	0.479324	−0.261119	0.612580	0.089*	
H18B	0.468461	−0.222714	0.672509	0.089*	
C19	0.40708 (14)	−0.35973 (19)	0.61840 (16)	0.0505 (8)	
H19	0.423336	−0.371444	0.584038	0.061*	
C20	0.35866 (16)	−0.3641 (2)	0.69164 (17)	0.0583 (9)	
H20	0.334635	−0.380078	0.717741	0.070*	
C21	0.38503 (17)	−0.3025 (2)	0.69669 (19)	0.0633 (10)	
H21	0.382814	−0.267919	0.726282	0.076*	
Zn2	0.23262 (2)	0.18410 (2)	0.66749 (2)	0.04854 (15)	
O5	0.18119 (10)	0.27391 (15)	0.64324 (13)	0.0624 (7)	
O6	0.17376 (13)	0.36649 (18)	0.57972 (18)	0.0891 (10)	
O7	0.30164 (10)	0.11551 (14)	0.65641 (12)	0.0589 (6)	
O8	0.37881 (13)	0.1195 (2)	0.61729 (18)	0.0929 (11)	
N6	0.27084 (11)	0.23577 (15)	0.60529 (12)	0.0458 (6)	
N7	0.25360 (12)	0.20574 (16)	0.75245 (12)	0.0497 (6)	
N8	0.26478 (13)	0.26157 (16)	0.83817 (12)	0.0542 (7)	
N9	0.17862 (11)	0.10751 (16)	0.65458 (12)	0.0479 (6)	
N10	0.10343 (12)	0.05191 (17)	0.64355 (13)	0.0539 (7)	
C22	0.33432 (14)	0.1431 (2)	0.62393 (18)	0.0576 (9)	
C23	0.31678 (13)	0.2106 (2)	0.59068 (15)	0.0510 (8)	
C24	0.34381 (17)	0.2462 (2)	0.54755 (18)	0.0656 (10)	
H24	0.376133	0.229334	0.537248	0.079*	
C25	0.32230 (19)	0.3054 (2)	0.52109 (19)	0.0700 (12)	
H25	0.339874	0.329162	0.492167	0.084*	
C26	0.27363 (17)	0.3315 (2)	0.53678 (17)	0.0621 (10)	
H26	0.258501	0.372057	0.518605	0.075*	
C27	0.24907 (14)	0.29458 (19)	0.58017 (15)	0.0499 (8)	
C28	0.19711 (15)	0.3142 (2)	0.60235 (18)	0.0569 (9)	
C29	0.12779 (14)	0.1150 (2)	0.64023 (15)	0.0514 (8)	
H29	0.111361	0.158047	0.629381	0.062*	
C30	0.18651 (14)	0.0363 (2)	0.66720 (16)	0.0543 (8)	
H30	0.218791	0.015381	0.678283	0.065*	
C31	0.14062 (17)	0.0014 (2)	0.66114 (19)	0.0599 (9)	
H31	0.135051	−0.047228	0.667554	0.072*	
C32	0.04643 (15)	0.0392 (3)	0.63624 (18)	0.0688 (11)	
H32A	0.039803	−0.006586	0.616551	0.083*	
H32B	0.030095	0.076346	0.610917	0.083*	
C33	0.01114 (16)	−0.0248 (2)	0.7231 (2)	0.0631 (10)	
H33	0.018907	−0.068264	0.705432	0.076*	
C34	0.02224 (13)	0.0390 (2)	0.69482 (16)	0.0532 (8)	
C35	0.01116 (14)	0.1028 (2)	0.72289 (18)	0.0582 (9)	
H35	0.018815	0.146225	0.705061	0.070*	
C36	0.2890 (2)	0.1678 (2)	0.78884 (18)	0.0718 (12)	
H36	0.305162	0.125229	0.778689	0.086*	
C37	0.2967 (2)	0.2017 (3)	0.84139 (18)	0.0782 (13)	
H37	0.319121	0.187619	0.873780	0.094*	
C38	0.23986 (14)	0.26132 (19)	0.78391 (15)	0.0484 (7)	
H38	0.215809	0.296024	0.770015	0.058*	
C39	0.2614 (2)	0.3162 (2)	0.88439 (17)	0.0656 (11)	
H39A	0.231811	0.347461	0.873945	0.079*	
H39B	0.292994	0.345273	0.886662	0.079*	
C40	0.29225 (19)	0.2940 (3)	0.99184 (18)	0.0733 (12)	
H40	0.320993	0.323274	0.986653	0.088*	
C41	0.25504 (16)	0.2821 (2)	0.94459 (15)	0.0555 (9)	
C42	0.21347 (19)	0.2377 (3)	0.95350 (18)	0.0760 (13)	
H42	0.188596	0.228885	0.921985	0.091*	
O9	0.06430 (16)	−0.0822 (2)	0.51541 (17)	0.1048 (12)	
O10	0.4733 (3)	0.1303 (7)	0.5644 (4)	0.190 (4)	0.75
O11	0.3252 (3)	−0.0187 (4)	0.7401 (5)	0.231 (4)	
O12	0.3802 (4)	0.0481 (8)	0.8545 (4)	0.218 (5)	0.75
O13	0.4461 (3)	0.1951 (4)	0.8617 (4)	0.205 (3)	
O14	0.3995 (2)	0.3330 (3)	0.8817 (3)	0.149 (2)	
O15	0.1232 (4)	0.3500 (5)	0.7219 (4)	0.110 (3)	0.5
O16	0.0825 (7)	0.3073 (9)	0.6926 (8)	0.201 (6)	0.5
O17	0.0290 (4)	0.4871 (6)	0.6036 (6)	0.142 (4)	0.5
O18	0.0628 (2)	0.3542 (4)	0.5554 (3)	0.164 (2)	
O19	0.0481 (2)	0.2013 (3)	0.5488 (2)	0.1211 (14)	

### catena-Poly[[[[(pyridine-2,6-dicarboxylato-κ3O,N,O')zinc(II)]-µ-1,4-bis(1H-imidazol-1-ylmethyl)benzene-κ2N1:N1'] hemi{bis[µ-1,4-bis(1H-imidazol-1-ylmethyl)benzene-κ2N1:N1']bis[(pyridine-2,6-dicarboxylato-κ3O,N,O')zinc(II)]}] nonahydrate] Atomic displacement parameters (Å^2^)

**Table:**

	*U*^11^	*U*^22^	*U*^33^	*U*^12^	*U*^13^	*U*^23^
Zn1	0.0652 (3)	0.0520 (3)	0.0485 (3)	−0.00767 (18)	0.0080 (2)	0.00127 (17)
O1	0.092 (2)	0.080 (2)	0.074 (2)	−0.0086 (18)	0.0374 (17)	−0.0050 (17)
O2	0.089 (3)	0.118 (3)	0.151 (4)	0.000 (2)	0.065 (3)	−0.027 (3)
O3	0.0668 (16)	0.0660 (17)	0.0540 (15)	0.0016 (13)	0.0156 (12)	0.0096 (12)
O4	0.095 (2)	0.0632 (17)	0.0742 (18)	−0.0235 (16)	0.0323 (16)	0.0026 (14)
N1	0.0531 (16)	0.0471 (15)	0.0553 (16)	−0.0076 (13)	0.0033 (13)	−0.0075 (13)
N2	0.073 (2)	0.0519 (17)	0.0481 (16)	−0.0067 (15)	0.0067 (14)	0.0044 (13)
N3	0.071 (2)	0.0533 (17)	0.0516 (17)	−0.0054 (15)	0.0004 (15)	0.0099 (13)
N4	0.0487 (15)	0.0421 (15)	0.0691 (19)	0.0052 (12)	0.0017 (14)	0.0068 (14)
N5	0.0580 (16)	0.0483 (15)	0.0453 (15)	0.0024 (13)	0.0115 (12)	0.0017 (12)
C1	0.065 (3)	0.083 (3)	0.090 (3)	−0.012 (2)	0.022 (2)	−0.032 (3)
C2	0.054 (2)	0.060 (2)	0.079 (3)	−0.0112 (18)	0.0023 (19)	−0.018 (2)
C3	0.068 (3)	0.064 (3)	0.118 (4)	0.008 (2)	−0.007 (3)	−0.018 (3)
C4	0.097 (4)	0.069 (3)	0.107 (4)	0.000 (3)	−0.011 (3)	0.014 (3)
C5	0.086 (3)	0.063 (3)	0.077 (3)	−0.010 (2)	−0.004 (2)	0.010 (2)
C6	0.067 (2)	0.0461 (19)	0.054 (2)	−0.0180 (16)	−0.0021 (16)	−0.0033 (15)
C7	0.065 (2)	0.054 (2)	0.0505 (19)	−0.0202 (17)	0.0068 (16)	−0.0048 (16)
C8	0.081 (3)	0.066 (2)	0.047 (2)	−0.007 (2)	0.0101 (19)	0.0053 (17)
C9	0.088 (3)	0.065 (2)	0.045 (2)	−0.009 (2)	0.001 (2)	0.0118 (17)
C10	0.073 (3)	0.054 (2)	0.047 (2)	−0.0024 (18)	0.0080 (17)	0.0044 (16)
C11	0.070 (3)	0.065 (3)	0.077 (3)	−0.003 (2)	−0.003 (2)	0.022 (2)
C12	0.066 (2)	0.049 (2)	0.065 (2)	−0.0064 (17)	0.0008 (18)	0.0106 (17)
C13	0.108 (4)	0.077 (3)	0.063 (3)	0.003 (3)	0.006 (2)	0.001 (2)
C14	0.105 (4)	0.062 (3)	0.088 (3)	0.018 (2)	0.021 (3)	−0.002 (2)
C15	0.054 (2)	0.048 (2)	0.083 (3)	−0.0074 (16)	0.0026 (19)	0.0085 (18)
C16	0.101 (3)	0.062 (3)	0.066 (3)	0.013 (2)	−0.013 (2)	0.004 (2)
C17	0.117 (4)	0.053 (2)	0.066 (3)	0.021 (2)	−0.012 (2)	−0.0015 (19)
C18	0.054 (2)	0.055 (2)	0.113 (4)	−0.0036 (18)	0.001 (2)	0.020 (2)
C19	0.0528 (19)	0.0494 (19)	0.0506 (18)	0.0053 (15)	0.0117 (15)	0.0042 (15)
C20	0.064 (2)	0.060 (2)	0.053 (2)	0.0032 (18)	0.0183 (17)	−0.0019 (17)
C21	0.068 (2)	0.059 (2)	0.064 (2)	0.0043 (19)	0.0114 (19)	−0.0105 (18)
Zn2	0.0533 (3)	0.0526 (3)	0.0404 (2)	−0.00723 (17)	0.00812 (17)	0.00025 (15)
O5	0.0595 (15)	0.0591 (16)	0.0701 (17)	0.0074 (12)	0.0144 (12)	0.0094 (13)
O6	0.083 (2)	0.0679 (19)	0.117 (3)	0.0150 (17)	0.0103 (19)	0.0342 (19)
O7	0.0580 (15)	0.0609 (15)	0.0586 (15)	0.0035 (12)	0.0091 (12)	−0.0005 (12)
O8	0.0667 (19)	0.095 (2)	0.121 (3)	0.0198 (18)	0.0283 (19)	0.004 (2)
N6	0.0493 (15)	0.0478 (15)	0.0405 (14)	−0.0089 (12)	0.0044 (11)	−0.0062 (11)
N7	0.0589 (16)	0.0487 (15)	0.0420 (14)	−0.0023 (13)	0.0083 (12)	−0.0009 (12)
N8	0.076 (2)	0.0500 (17)	0.0369 (14)	0.0002 (14)	0.0074 (13)	−0.0006 (12)
N9	0.0486 (15)	0.0531 (16)	0.0429 (14)	−0.0039 (12)	0.0095 (11)	0.0039 (12)
N10	0.0503 (16)	0.0661 (19)	0.0460 (15)	−0.0104 (14)	0.0077 (12)	0.0019 (13)
C22	0.050 (2)	0.061 (2)	0.062 (2)	0.0030 (17)	0.0084 (16)	−0.0160 (18)
C23	0.0468 (18)	0.060 (2)	0.0471 (18)	−0.0108 (15)	0.0092 (14)	−0.0131 (16)
C24	0.062 (2)	0.076 (3)	0.061 (2)	−0.020 (2)	0.0190 (18)	−0.010 (2)
C25	0.087 (3)	0.074 (3)	0.052 (2)	−0.035 (2)	0.022 (2)	−0.0064 (19)
C26	0.082 (3)	0.051 (2)	0.054 (2)	−0.0253 (19)	0.0054 (19)	0.0018 (16)
C27	0.062 (2)	0.0435 (17)	0.0433 (17)	−0.0111 (15)	−0.0006 (15)	−0.0045 (14)
C28	0.057 (2)	0.049 (2)	0.065 (2)	−0.0018 (16)	0.0048 (17)	0.0013 (17)
C29	0.0517 (19)	0.055 (2)	0.0476 (18)	−0.0014 (16)	0.0043 (14)	0.0060 (15)
C30	0.053 (2)	0.055 (2)	0.056 (2)	0.0025 (16)	0.0131 (16)	0.0075 (16)
C31	0.066 (2)	0.053 (2)	0.063 (2)	−0.0064 (17)	0.0173 (19)	0.0013 (17)
C32	0.047 (2)	0.100 (3)	0.058 (2)	−0.018 (2)	−0.0008 (16)	−0.003 (2)
C33	0.057 (2)	0.057 (2)	0.077 (3)	−0.0044 (17)	0.0147 (18)	−0.0129 (19)
C34	0.0375 (16)	0.065 (2)	0.057 (2)	−0.0077 (15)	−0.0007 (14)	0.0008 (17)
C35	0.0463 (18)	0.056 (2)	0.072 (2)	−0.0033 (16)	−0.0017 (16)	0.0110 (18)
C36	0.100 (3)	0.063 (2)	0.052 (2)	0.024 (2)	0.000 (2)	−0.0046 (18)
C37	0.109 (4)	0.078 (3)	0.045 (2)	0.030 (3)	−0.009 (2)	−0.001 (2)
C38	0.0548 (19)	0.0474 (18)	0.0437 (17)	−0.0054 (15)	0.0078 (14)	0.0071 (14)
C39	0.098 (3)	0.052 (2)	0.047 (2)	−0.008 (2)	0.0097 (19)	−0.0062 (16)
C40	0.082 (3)	0.086 (3)	0.052 (2)	−0.033 (2)	0.007 (2)	−0.005 (2)
C41	0.075 (2)	0.052 (2)	0.0404 (17)	−0.0023 (17)	0.0066 (16)	−0.0087 (15)
C42	0.082 (3)	0.099 (3)	0.045 (2)	−0.027 (3)	−0.0087 (19)	−0.003 (2)
O9	0.106 (3)	0.128 (3)	0.085 (2)	0.007 (2)	0.036 (2)	0.011 (2)
O10	0.076 (4)	0.329 (11)	0.168 (7)	−0.017 (6)	0.033 (4)	−0.016 (7)
O11	0.139 (6)	0.194 (7)	0.376 (14)	0.006 (5)	0.116 (7)	−0.021 (7)
O12	0.197 (9)	0.324 (15)	0.133 (7)	0.067 (10)	0.018 (6)	−0.005 (8)
O13	0.211 (7)	0.167 (6)	0.234 (9)	0.048 (5)	0.006 (6)	0.035 (6)
O14	0.157 (5)	0.133 (4)	0.147 (5)	0.014 (4)	−0.047 (4)	−0.015 (3)
O15	0.143 (7)	0.091 (5)	0.102 (6)	0.039 (5)	0.059 (5)	0.024 (4)
O16	0.215 (13)	0.217 (13)	0.176 (11)	0.022 (11)	0.040 (10)	0.081 (10)
O17	0.094 (6)	0.137 (8)	0.197 (12)	0.008 (6)	0.013 (7)	−0.024 (8)
O18	0.114 (4)	0.163 (5)	0.217 (7)	−0.007 (4)	0.029 (4)	0.004 (5)
O19	0.134 (4)	0.116 (3)	0.113 (3)	0.020 (3)	0.005 (3)	0.005 (3)

### catena-Poly[[[[(pyridine-2,6-dicarboxylato-κ3O,N,O')zinc(II)]-µ-1,4-bis(1H-imidazol-1-ylmethyl)benzene-κ2N1:N1'] hemi{bis[µ-1,4-bis(1H-imidazol-1-ylmethyl)benzene-κ2N1:N1']bis[(pyridine-2,6-dicarboxylato-κ3O,N,O')zinc(II)]}] nonahydrate] Geometric parameters (Å, º)

**Table:**

Zn1—O1	2.208 (3)	Zn2—O7	2.209 (3)
Zn1—O3	2.151 (3)	Zn2—N6	2.010 (3)
Zn1—N1	2.014 (3)	Zn2—N7	1.984 (3)
Zn1—N2	1.984 (3)	Zn2—N9	1.992 (3)
Zn1—N5^i^	1.990 (3)	O5—C28	1.277 (5)
O1—C1	1.267 (6)	O6—C28	1.232 (5)
O2—C1	1.225 (6)	O7—C22	1.260 (5)
O3—C7	1.257 (5)	O8—C22	1.239 (5)
O4—C7	1.241 (4)	N6—C23	1.329 (4)
N1—C2	1.332 (5)	N6—C27	1.335 (5)
N1—C6	1.324 (5)	N7—C36	1.370 (5)
N2—C8	1.383 (5)	N7—C38	1.316 (5)
N2—C10	1.318 (5)	N8—C37	1.380 (5)
N3—C9	1.362 (6)	N8—C38	1.332 (4)
N3—C10	1.333 (5)	N8—C39	1.461 (5)
N3—C11	1.482 (6)	N9—C29	1.323 (5)
N4—C18	1.470 (5)	N9—C30	1.368 (5)
N4—C19	1.324 (5)	N10—C29	1.335 (5)
N4—C21	1.373 (5)	N10—C31	1.375 (5)
N5—C19	1.308 (4)	N10—C32	1.474 (5)
N5—C20	1.366 (5)	C22—C23	1.514 (6)
C1—C2	1.506 (7)	C23—C24	1.399 (5)
C2—C3	1.401 (7)	C24—H24	0.9300
C3—H3	0.9300	C24—C25	1.350 (7)
C3—C4	1.377 (8)	C25—H25	0.9300
C4—H4	0.9300	C25—C26	1.406 (7)
C4—C5	1.377 (8)	C26—H26	0.9300
C5—H5	0.9300	C26—C27	1.382 (5)
C5—C6	1.381 (6)	C27—C28	1.500 (5)
C6—C7	1.518 (6)	C29—H29	0.9300
C8—H8	0.9300	C30—H30	0.9300
C8—C9	1.345 (7)	C30—C31	1.340 (6)
C9—H9	0.9300	C31—H31	0.9300
C10—H10	0.9300	C32—H32A	0.9700
C11—H11A	0.9700	C32—H32B	0.9700
C11—H11B	0.9700	C32—C34	1.496 (6)
C11—C12	1.498 (5)	C33—C33^ii^	1.373 (8)
C12—C13	1.385 (6)	C33—H33	0.9300
C12—C17	1.368 (6)	C33—C34	1.388 (6)
C13—H13	0.9300	C34—C35	1.385 (6)
C13—C14	1.382 (7)	C35—C35^ii^	1.383 (8)
C14—H14	0.9300	C35—H35	0.9300
C14—C15	1.362 (7)	C36—H36	0.9300
C15—C16	1.369 (6)	C36—C37	1.340 (6)
C15—C18	1.500 (6)	C37—H37	0.9300
C16—H16	0.9300	C38—H38	0.9300
C16—C17	1.393 (6)	C39—H39A	0.9700
C17—H17	0.9300	C39—H39B	0.9700
C18—H18A	0.9700	C39—C41	1.515 (5)
C18—H18B	0.9700	C40—H40	0.9300
C19—H19	0.9300	C40—C41	1.389 (6)
C20—H20	0.9300	C40—C42^iii^	1.380 (6)
C20—C21	1.332 (6)	C41—C42	1.373 (6)
C21—H21	0.9300	C42—H42	0.9300
Zn2—O5	2.172 (3)		
			
O3—Zn1—O1	152.50 (13)	O5—Zn2—O7	153.06 (10)
N1—Zn1—O1	75.70 (13)	N6—Zn2—O5	76.93 (11)
N1—Zn1—O3	77.05 (11)	N6—Zn2—O7	76.35 (11)
N2—Zn1—O1	98.27 (14)	N7—Zn2—O5	101.49 (12)
N2—Zn1—O3	94.35 (13)	N7—Zn2—O7	93.77 (11)
N2—Zn1—N1	125.19 (12)	N7—Zn2—N6	117.44 (11)
N2—Zn1—N5^i^	114.81 (12)	N7—Zn2—N9	114.82 (12)
N5^i^—Zn1—O1	97.97 (12)	N9—Zn2—O5	96.62 (12)
N5^i^—Zn1—O3	98.73 (11)	N9—Zn2—O7	96.92 (11)
N5^i^—Zn1—N1	119.99 (12)	N9—Zn2—N6	127.60 (11)
C1—O1—Zn1	114.9 (3)	C28—O5—Zn2	114.4 (2)
C7—O3—Zn1	115.2 (2)	C22—O7—Zn2	113.6 (2)
C2—N1—Zn1	119.7 (3)	C23—N6—Zn2	119.2 (2)
C6—N1—Zn1	118.2 (3)	C23—N6—C27	122.3 (3)
C6—N1—C2	122.0 (4)	C27—N6—Zn2	118.5 (2)
C8—N2—Zn1	130.5 (3)	C36—N7—Zn2	126.0 (3)
C10—N2—Zn1	123.1 (3)	C38—N7—Zn2	127.8 (2)
C10—N2—C8	105.9 (3)	C38—N7—C36	106.1 (3)
C9—N3—C11	127.1 (4)	C37—N8—C39	126.3 (3)
C10—N3—C9	107.4 (4)	C38—N8—C37	106.6 (3)
C10—N3—C11	125.3 (4)	C38—N8—C39	127.1 (3)
C19—N4—C18	126.4 (4)	C29—N9—Zn2	128.2 (3)
C19—N4—C21	107.0 (3)	C29—N9—C30	106.2 (3)
C21—N4—C18	126.5 (4)	C30—N9—Zn2	125.1 (2)
C19—N5—Zn1^i^	128.6 (2)	C29—N10—C31	107.6 (3)
C19—N5—C20	105.9 (3)	C29—N10—C32	126.7 (4)
C20—N5—Zn1^i^	125.5 (2)	C31—N10—C32	125.4 (3)
O1—C1—C2	115.4 (4)	O7—C22—C23	116.1 (3)
O2—C1—O1	127.0 (5)	O8—C22—O7	125.6 (4)
O2—C1—C2	117.5 (5)	O8—C22—C23	118.4 (4)
N1—C2—C1	114.1 (4)	N6—C23—C22	114.0 (3)
N1—C2—C3	119.7 (4)	N6—C23—C24	119.7 (4)
C3—C2—C1	126.2 (4)	C24—C23—C22	126.3 (3)
C2—C3—H3	120.8	C23—C24—H24	120.5
C4—C3—C2	118.4 (5)	C25—C24—C23	119.1 (4)
C4—C3—H3	120.8	C25—C24—H24	120.5
C3—C4—H4	119.8	C24—C25—H25	119.6
C5—C4—C3	120.5 (5)	C24—C25—C26	120.7 (4)
C5—C4—H4	119.8	C26—C25—H25	119.6
C4—C5—H5	120.8	C25—C26—H26	121.2
C4—C5—C6	118.4 (5)	C27—C26—C25	117.6 (4)
C6—C5—H5	120.8	C27—C26—H26	121.2
N1—C6—C5	121.0 (4)	N6—C27—C26	120.6 (4)
N1—C6—C7	113.8 (3)	N6—C27—C28	114.3 (3)
C5—C6—C7	125.2 (4)	C26—C27—C28	125.1 (4)
O3—C7—C6	115.5 (3)	O5—C28—C27	115.7 (3)
O4—C7—O3	126.8 (4)	O6—C28—O5	126.2 (4)
O4—C7—C6	117.8 (4)	O6—C28—C27	118.1 (4)
N2—C8—H8	125.7	N9—C29—N10	110.4 (3)
C9—C8—N2	108.6 (4)	N9—C29—H29	124.8
C9—C8—H8	125.7	N10—C29—H29	124.8
N3—C9—H9	126.5	N9—C30—H30	125.2
C8—C9—N3	107.0 (4)	C31—C30—N9	109.6 (3)
C8—C9—H9	126.5	C31—C30—H30	125.2
N2—C10—N3	111.0 (4)	N10—C31—H31	126.9
N2—C10—H10	124.5	C30—C31—N10	106.2 (3)
N3—C10—H10	124.5	C30—C31—H31	126.9
N3—C11—H11A	109.0	N10—C32—H32A	109.2
N3—C11—H11B	109.0	N10—C32—H32B	109.2
N3—C11—C12	112.8 (3)	N10—C32—C34	112.0 (3)
H11A—C11—H11B	107.8	H32A—C32—H32B	107.9
C12—C11—H11A	109.0	C34—C32—H32A	109.2
C12—C11—H11B	109.0	C34—C32—H32B	109.2
C13—C12—C11	122.0 (4)	C33^ii^—C33—H33	119.5
C17—C12—C11	119.7 (4)	C33^ii^—C33—C34	121.0 (2)
C17—C12—C13	118.3 (4)	C34—C33—H33	119.5
C12—C13—H13	119.8	C33—C34—C32	121.2 (4)
C14—C13—C12	120.5 (5)	C35—C34—C32	120.7 (4)
C14—C13—H13	119.8	C35—C34—C33	118.1 (4)
C13—C14—H14	119.4	C34—C35—H35	119.6
C15—C14—C13	121.3 (4)	C35^ii^—C35—C34	120.9 (2)
C15—C14—H14	119.4	C35^ii^—C35—H35	119.6
C14—C15—C16	118.5 (4)	N7—C36—H36	125.4
C14—C15—C18	120.9 (4)	C37—C36—N7	109.1 (4)
C16—C15—C18	120.6 (4)	C37—C36—H36	125.4
C15—C16—H16	119.5	N8—C37—H37	126.6
C15—C16—C17	121.0 (4)	C36—C37—N8	106.8 (4)
C17—C16—H16	119.5	C36—C37—H37	126.6
C12—C17—C16	120.5 (4)	N7—C38—N8	111.4 (3)
C12—C17—H17	119.8	N7—C38—H38	124.3
C16—C17—H17	119.8	N8—C38—H38	124.3
N4—C18—C15	112.6 (3)	N8—C39—H39A	109.4
N4—C18—H18A	109.1	N8—C39—H39B	109.4
N4—C18—H18B	109.1	N8—C39—C41	110.9 (3)
C15—C18—H18A	109.1	H39A—C39—H39B	108.0
C15—C18—H18B	109.1	C41—C39—H39A	109.4
H18A—C18—H18B	107.8	C41—C39—H39B	109.4
N4—C19—H19	124.3	C41—C40—H40	120.0
N5—C19—N4	111.3 (3)	C42^iii^—C40—H40	120.0
N5—C19—H19	124.3	C42^iii^—C40—C41	119.9 (4)
N5—C20—H20	125.3	C40—C41—C39	120.1 (4)
C21—C20—N5	109.5 (3)	C42—C41—C39	121.3 (4)
C21—C20—H20	125.3	C42—C41—C40	118.6 (4)
N4—C21—H21	126.8	C40^iii^—C42—H42	119.3
C20—C21—N4	106.3 (3)	C41—C42—C40^iii^	121.5 (4)
C20—C21—H21	126.8	C41—C42—H42	119.3
			
Zn1—O1—C1—O2	−179.6 (4)	C21—N4—C19—N5	0.1 (4)
Zn1—O1—C1—C2	−1.1 (5)	Zn2—O5—C28—O6	177.0 (4)
Zn1—O3—C7—O4	177.0 (3)	Zn2—O5—C28—C27	−2.4 (4)
Zn1—O3—C7—C6	−4.0 (4)	Zn2—O7—C22—O8	170.2 (4)
Zn1—N1—C2—C1	5.5 (4)	Zn2—O7—C22—C23	−9.8 (4)
Zn1—N1—C2—C3	−175.0 (3)	Zn2—N6—C23—C22	0.7 (4)
Zn1—N1—C6—C5	174.9 (3)	Zn2—N6—C23—C24	−179.9 (3)
Zn1—N1—C6—C7	−5.5 (4)	Zn2—N6—C27—C26	−179.3 (3)
Zn1—N2—C8—C9	−170.5 (3)	Zn2—N6—C27—C28	−0.4 (4)
Zn1—N2—C10—N3	172.0 (2)	Zn2—N7—C36—C37	174.6 (3)
Zn1^i^—N5—C19—N4	177.9 (2)	Zn2—N7—C38—N8	−174.8 (2)
Zn1^i^—N5—C20—C21	−177.9 (3)	Zn2—N9—C29—N10	−171.5 (2)
O1—C1—C2—N1	−2.6 (5)	Zn2—N9—C30—C31	171.4 (3)
O1—C1—C2—C3	177.9 (4)	O7—C22—C23—N6	6.6 (5)
O2—C1—C2—N1	176.1 (4)	O7—C22—C23—C24	−172.8 (3)
O2—C1—C2—C3	−3.4 (7)	O8—C22—C23—N6	−173.4 (4)
N1—C2—C3—C4	−0.4 (7)	O8—C22—C23—C24	7.2 (6)
N1—C6—C7—O3	6.3 (5)	N6—C23—C24—C25	−0.6 (5)
N1—C6—C7—O4	−174.7 (3)	N6—C27—C28—O5	2.0 (5)
N2—C8—C9—N3	−1.4 (5)	N6—C27—C28—O6	−177.5 (4)
N3—C11—C12—C13	94.6 (5)	N7—C36—C37—N8	0.9 (6)
N3—C11—C12—C17	−87.5 (5)	N8—C39—C41—C40	119.8 (4)
N5—C20—C21—N4	−0.4 (5)	N8—C39—C41—C42	−58.7 (6)
C1—C2—C3—C4	179.0 (5)	N9—C30—C31—N10	0.8 (4)
C2—N1—C6—C5	−1.4 (5)	N10—C32—C34—C33	−99.3 (4)
C2—N1—C6—C7	178.2 (3)	N10—C32—C34—C35	79.5 (5)
C2—C3—C4—C5	−0.3 (8)	C22—C23—C24—C25	178.8 (4)
C3—C4—C5—C6	0.2 (8)	C23—N6—C27—C26	0.9 (5)
C4—C5—C6—N1	0.7 (6)	C23—N6—C27—C28	179.8 (3)
C4—C5—C6—C7	−178.9 (4)	C23—C24—C25—C26	0.5 (6)
C5—C6—C7—O3	−174.2 (4)	C24—C25—C26—C27	0.3 (6)
C5—C6—C7—O4	4.9 (5)	C25—C26—C27—N6	−1.0 (5)
C6—N1—C2—C1	−178.3 (3)	C25—C26—C27—C28	−179.7 (3)
C6—N1—C2—C3	1.2 (6)	C26—C27—C28—O5	−179.2 (3)
C8—N2—C10—N3	−0.3 (4)	C26—C27—C28—O6	1.3 (6)
C9—N3—C10—N2	−0.5 (4)	C27—N6—C23—C22	−179.5 (3)
C9—N3—C11—C12	67.4 (6)	C27—N6—C23—C24	−0.1 (5)
C10—N2—C8—C9	1.1 (4)	C29—N9—C30—C31	−0.7 (4)
C10—N3—C9—C8	1.1 (4)	C29—N10—C31—C30	−0.6 (4)
C10—N3—C11—C12	−118.4 (4)	C29—N10—C32—C34	−94.2 (5)
C11—N3—C9—C8	176.2 (4)	C30—N9—C29—N10	0.3 (4)
C11—N3—C10—N2	−175.7 (3)	C31—N10—C29—N9	0.2 (4)
C11—C12—C13—C14	176.9 (5)	C31—N10—C32—C34	78.7 (5)
C11—C12—C17—C16	−178.0 (5)	C32—N10—C29—N9	174.1 (3)
C12—C13—C14—C15	1.0 (8)	C32—N10—C31—C30	−174.6 (3)
C13—C12—C17—C16	0.0 (7)	C32—C34—C35—C35^ii^	−179.9 (4)
C13—C14—C15—C16	0.1 (8)	C33^ii^—C33—C34—C32	−179.9 (5)
C13—C14—C15—C18	−179.4 (5)	C33^ii^—C33—C34—C35	1.3 (7)
C14—C15—C16—C17	−1.2 (7)	C33—C34—C35—C35^ii^	−1.1 (6)
C14—C15—C18—N4	−86.7 (6)	C36—N7—C38—N8	0.6 (4)
C15—C16—C17—C12	1.1 (8)	C37—N8—C38—N7	0.0 (4)
C16—C15—C18—N4	93.8 (5)	C37—N8—C39—C41	−48.9 (6)
C17—C12—C13—C14	−1.0 (8)	C38—N7—C36—C37	−0.9 (5)
C18—N4—C19—N5	−178.8 (3)	C38—N8—C37—C36	−0.5 (5)
C18—N4—C21—C20	179.1 (4)	C38—N8—C39—C41	134.6 (4)
C18—C15—C16—C17	178.3 (4)	C39—N8—C37—C36	−177.6 (4)
C19—N4—C18—C15	95.0 (5)	C39—N8—C38—N7	177.0 (3)
C19—N4—C21—C20	0.2 (4)	C39—C41—C42—C40^iii^	179.4 (5)
C19—N5—C20—C21	0.4 (4)	C40—C41—C42—C40^iii^	0.9 (8)
C20—N5—C19—N4	−0.3 (4)	C42^iii^—C40—C41—C39	−179.5 (4)
C21—N4—C18—C15	−83.8 (5)	C42^iii^—C40—C41—C42	−0.9 (8)

Symmetry codes: (i) −*x*+1/2, −*y*−1/2, −*z*+1; (ii) −*x*, *y*, −*z*+3/2; (iii) −*x*+1/2, −*y*+1/2, −*z*+2.

### catena-Poly[[[[(pyridine-2,6-dicarboxylato-κ3O,N,O')zinc(II)]-µ-1,4-bis(1H-imidazol-1-ylmethyl)benzene-κ2N1:N1'] hemi{bis[µ-1,4-bis(1H-imidazol-1-ylmethyl)benzene-κ2N1:N1']bis[(pyridine-2,6-dicarboxylato-κ3O,N,O')zinc(II)]}] nonahydrate] Hydrogen-bond geometry (Å, º)

*Cg*3, *Cg*10 and *Cg*11 are the centroids of the N7/C36/C37/N8/C38,N2/C8/C9/N3/C10 and N4/C20/C21/N5/C19 rings, respectively.

**Table:**

*D*—H···*A*	*D*—H	H···*A*	*D*···*A*	*D*—H···*A*
C18—H18*A*···O19^iv^	0.97	2.45	3.421 (7)	174
C19—H19···O9^i^	0.93	2.44	3.328 (5)	160
C21—H21···O15^v^	0.93	2.50	3.394 (10)	162
C21—H21···O16^v^	0.93	2.41	3.278 (18)	154
C29—H29···O19	0.93	2.47	3.213 (6)	137
C30—H30···O4^vi^	0.93	2.53	3.149 (5)	124
C38—H38···O4^vii^	0.93	2.58	2.988 (5)	107
C5—H5···*Cg*3^vii^	0.93	2.81	3.624 (5)	146
C26—H26···*Cg*10^vii^	0.93	2.61	3.443 (4)	150
C42—H42···*Cg*11^vi^	0.93	2.93	3.818 (5)	161

Symmetry codes: (i) −*x*+1/2, −*y*−1/2, −*z*+1; (iv) *x*+1/2, *y*−1/2, *z*; (v) −*x*+1/2, *y*−1/2, −*z*+3/2; (vi) *x*, −*y*, *z*+1/2; (vii) −*x*+1/2, −*y*+1/2, −*z*+1.
