# Supplementary material for: Crystal structure and characterization of a new chain-like polyrotaxane zinc(II) coordination polymer with mixed pyridine-2,6-di­carboxyl­ate and 1,4-bis­(1H-imidazol-1-ylmeth­yl)benzene ligands
Source: Acta Crystallogr E Crystallogr Commun. 2025 May 30;81(Pt 6):559–64. doi: 10.1107/S2056989025004608 (PMC12142422; doi:10.1107/S2056989025004608)
Supplement: Supplementary file 2 [file e-81-00559-sup4.pdf]

Supporting Information

Crystal structure and characterization of a new one-dimensional polyrotaxane zinc(II) coordination polymer with mixed pyridine-2,6-dicarboxylate and 1,4-bis(imidazole-1-ly-methyl)benzene ligands

Chanikarn Kummuang [a], Kittipong Chainok [b], Nanthawat Wannarit [a,b,*]

[a] Department of Chemistry, Faculty of Science and Technology, Thammasat University, Pathum Thani, 12121, Thailand

[b] Thammasat University Research Unit in Multifunctional Crystalline Materials and Applications (TU-MCMA), Faculty of Science and Technology, Thammasat University, Pathum Thani 12121, Thailand

List of Figures

Fig. S1 Views of five-coordinate geometry for Zn(II) ions with the τ parameter of the title compound.

Fig. S2 (a) Top and (b) side views of *gauche*-conformation and (c) Newmann projection representation of the bix ligand in the dinuclear Zn(1) macrocyclic unit (Symmetry code (i) = -x+1/2, -y-1/2, -z+1).

Fig. S3 (a) Top and (b) side views of *trans*-conformation and (c) Newmann projection representation of the first bix ligand in the 1D zigzag chain-like Zn(2) unit (Symmetry code (ii) = -x+1/2, -y+1/2, -z+2).

Fig. S4 (a) Top and (b) side views of *trans*-conformation and (c) Newmann projection representation of the second bix ligand in the 1D zigzag chain-like Zn(2) unit (Symmetry code (iii) = -x, +y, -z+3/2).

Fig. S5 The hydrogen bonding C-H···O intermolecular interactions between C-H group of imidazole ring on the bix ligands and oxygen atoms of the carboxylate group of 2,6-PDC ligands (Symmetry codes (iv) = *x*, -*y*, *z*+1/2 and (v) = -*x*+1/2, -*y*+1/2, -*z*+1).

Fig. S6 The hydrogen bonding C-H···O intermolecular interactions between the C-H groups of both the imidazole ring and the -CH_2 group on the bix ligands and the lattice water molecules (Symmetry codes (i) -x+1/2, -y-1/2, -z+1; (ii) x+1/2, y-1/2, z; (iii) -x+1/2, y-1/2).

Fig. S7 View of the π···π stacking interactions between the pyridine rings of 2,6-PDC ligands in the adjacent 1D zigzag chains and the dinuclear units (Symmetry code (i) = 1/2-x, 1/2-y, 1-z).

Fig. S8 The C-H···π interactions between the C-H group of phenyl ring of bix and imidazole ring of bix ligands and aromatic imidazole rings in the title compound (Symmetry codes (iv) = *x*, -*y*, *z*+1/2 and (v) = -*x*+1/2, -*y*+1/2, -z+1).

Fig. S9 The 3D crystal packing of the title compound in crystallographic [001] direction.

Fig. S10 IR spectrum of the title compound.

Fig. S11 Powder XRD patterns of the title compound.

Fig. S12 TGA curve of the title compound.

Scheme S1

Table S1 Geometrical parameters describing the conformations of bix ligands in the title compound (see also **Scheme S1**)

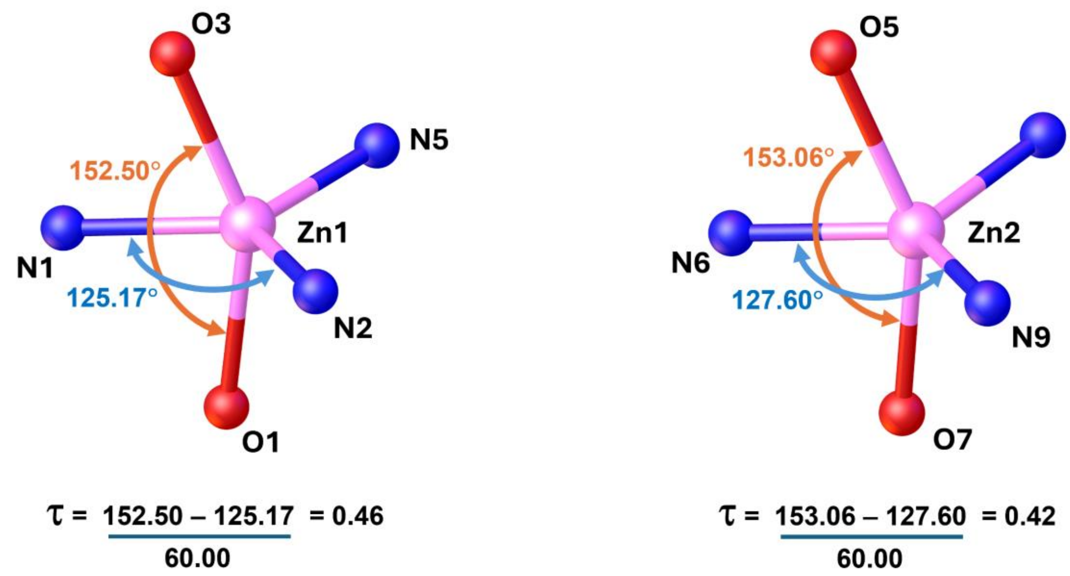

Fig. S1 Views of five-coordinate geometry for Zn(II) ions with the τ parameter of the title compound.

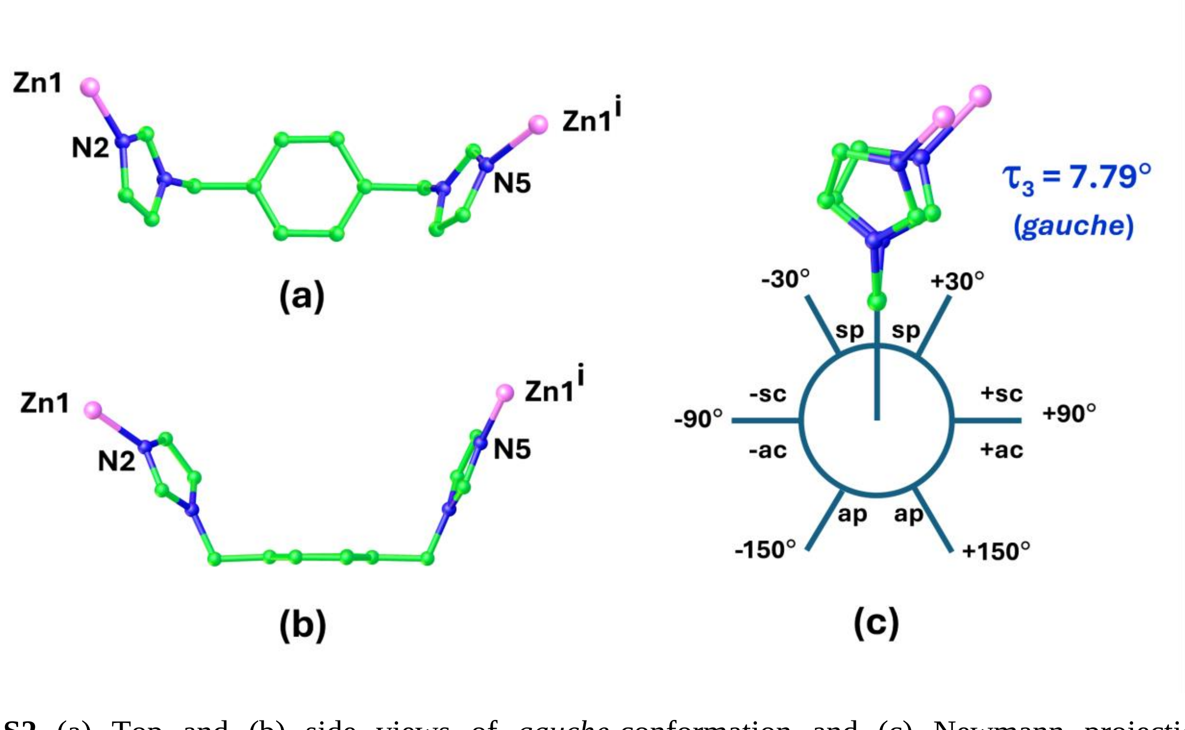

Fig. S2 (a) Top and (b) side views of *gauche*-conformation and (c) Newmann projection representation of the bix ligand in the dinuclear Zn(1) macrocyclic unit (Symmetry code (i) = -x+1/2, -y-1/2, -z+1).

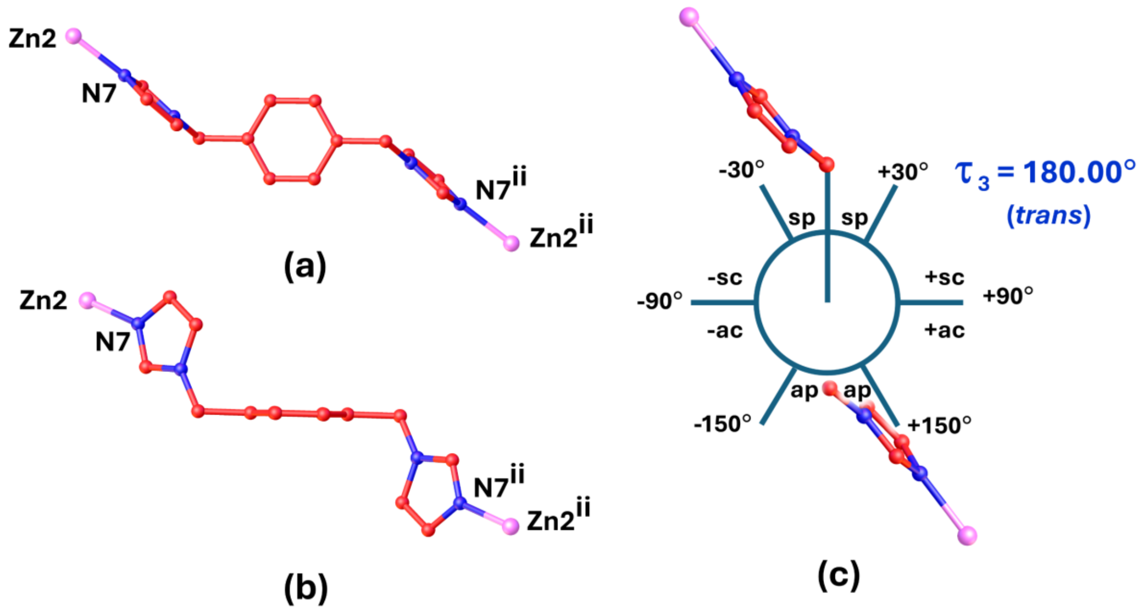

Fig. S3 (a) Top and (b) side views of *trans*-conformation and (c) Newmann projection representation of the first bix ligand in the 1D zigzag chain-like Zn(2) unit (Symmetry code (ii) = -x+1/2, -y+1/2, -z+2).

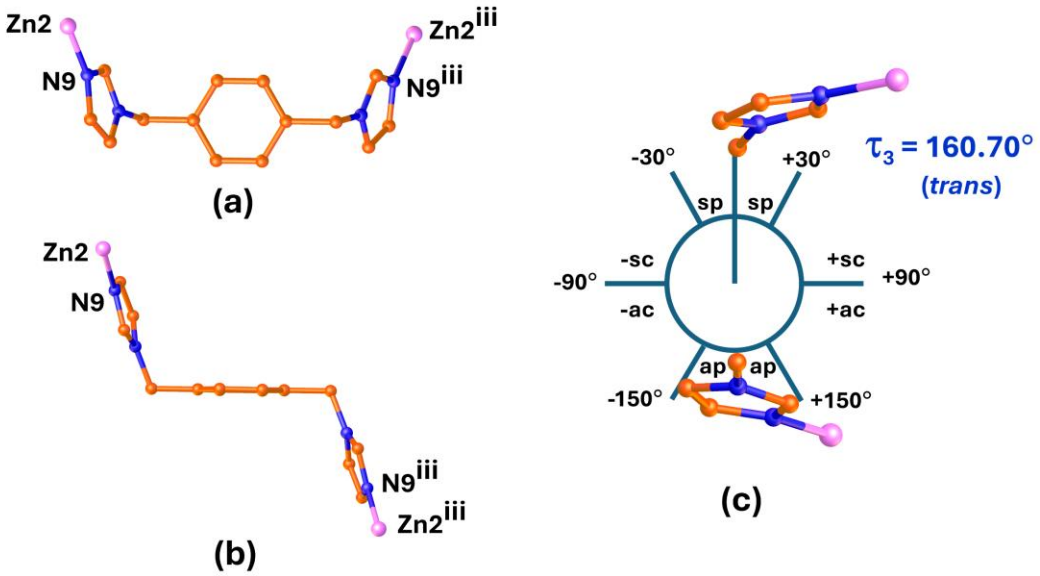

Fig. S4 (a) Top and (b) side views of *trans*-conformation and (c) Newmann projection representation of the second bix ligand in the 1D zigzag chain-like Zn(2) unit (Symmetry code (iii) = -x, +y, -z+3/2).

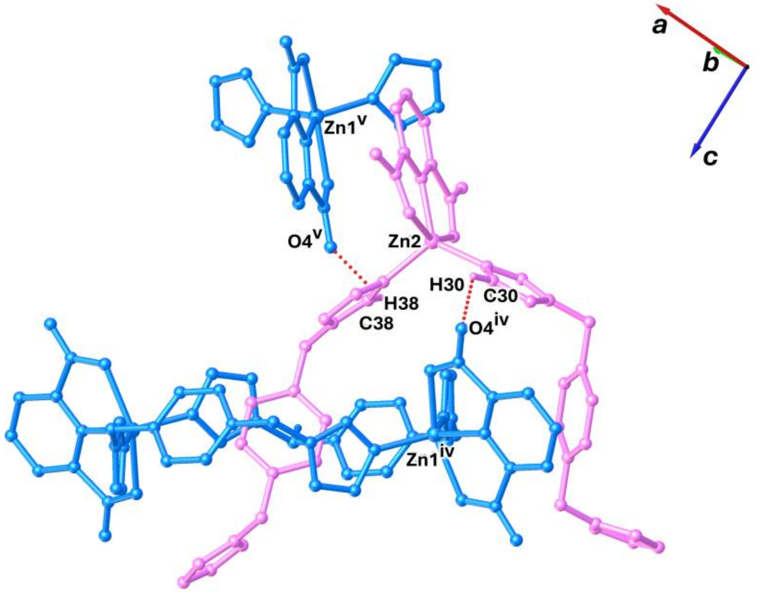

Fig. S5 The hydrogen bonding C-H···O intermolecular interactions between C-H group of imidazole ring on the bix ligands and oxygen atoms of the carboxylate group of 2,6-PDC ligands (Symmetry codes (iv) = *x*, -*y*, *z*+1/2 and (v) = -*x*+1/2, -*y*+1/2, -*z*+1).

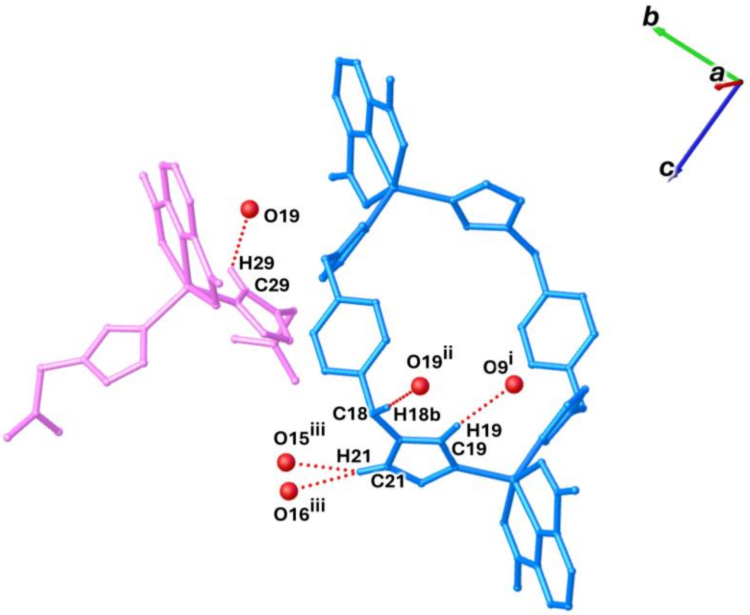

Fig. S6 The hydrogen bonding C-H···O intermolecular interactions between the C-H groups of both the imidazole ring and the -CH_2 group on the bix ligands and the lattice water molecules (Symmetry codes (i) -x+1/2, -y-1/2, -z+1; (ii) x+1/2, y-1/2, z; (iii) -x+1/2, y-1/2).

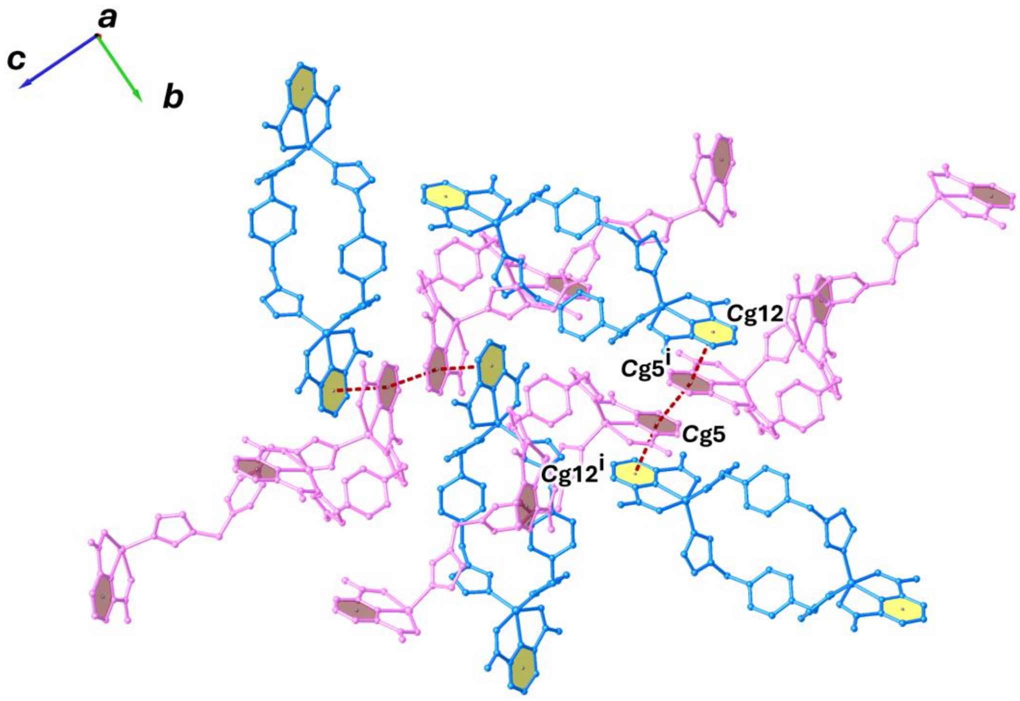

Fig. S7 View of the π···π stacking interactions between the pyridine rings of 2,6-PDC ligands in the adjacent 1D zigzag chains and the dinuclear units (Symmetry code (i) = 1/2-x, 1/2-y, 1-z).

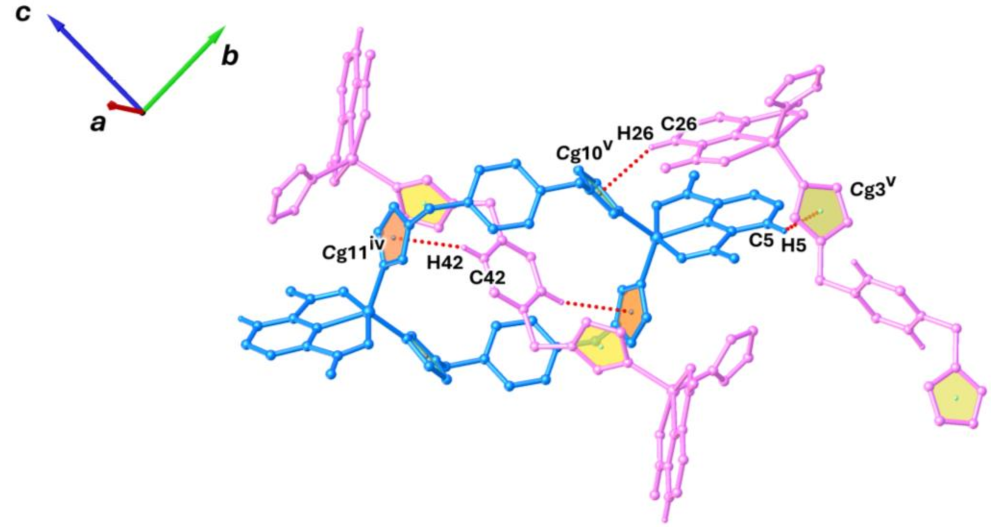

Fig. S8 The C-H···π interactions between the C-H group of phenyl ring of bix and imidazole ring of bix ligands and aromatic imidazole rings in the title compound (Symmetry codes (iv) = *x*, -*y*, *z*+1/2 and (v) = -*x*+1/2, -*y*+1/2, -*z*+1).

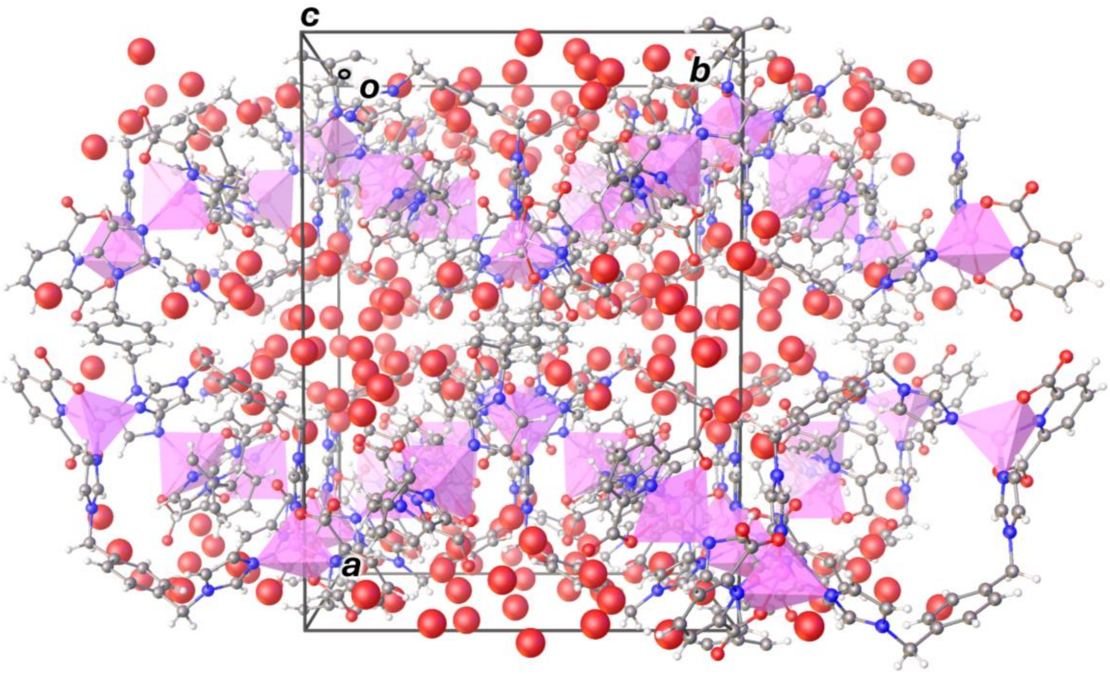

Fig. S9 The 3D crystal packing of the title compound in crystallographic [001] direction.

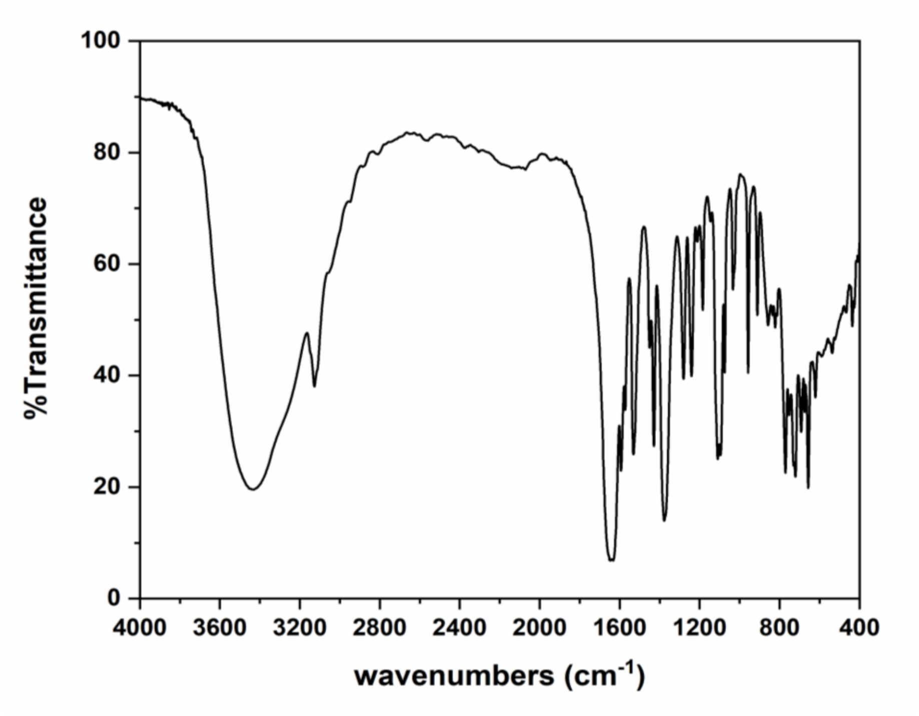

Fig. S10 FTIR spectrum of the title compound.

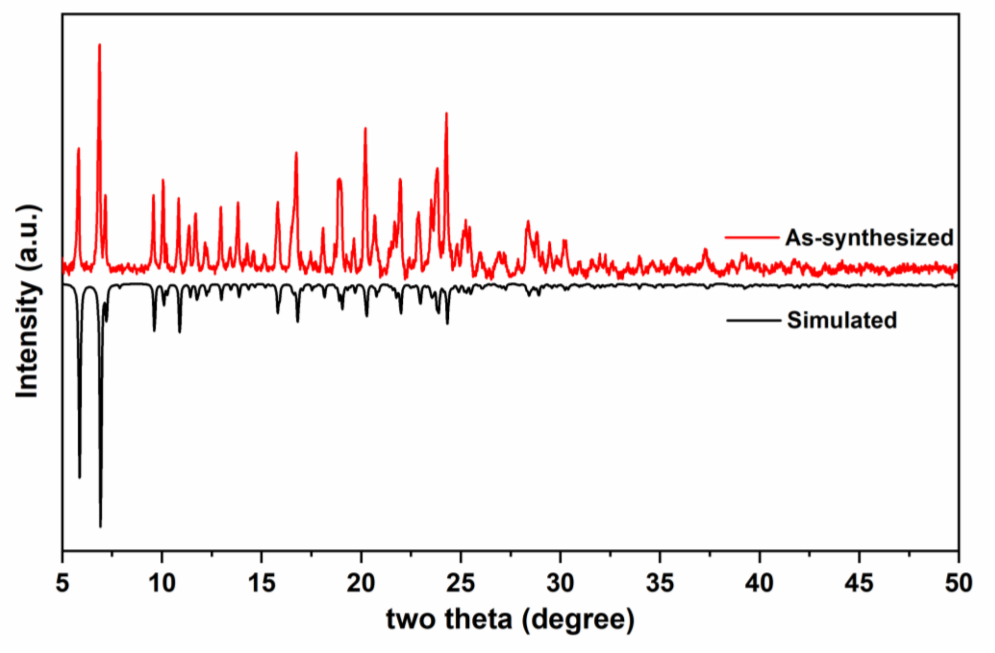

Fig. S11 Powder XRD patterns of the title compound.

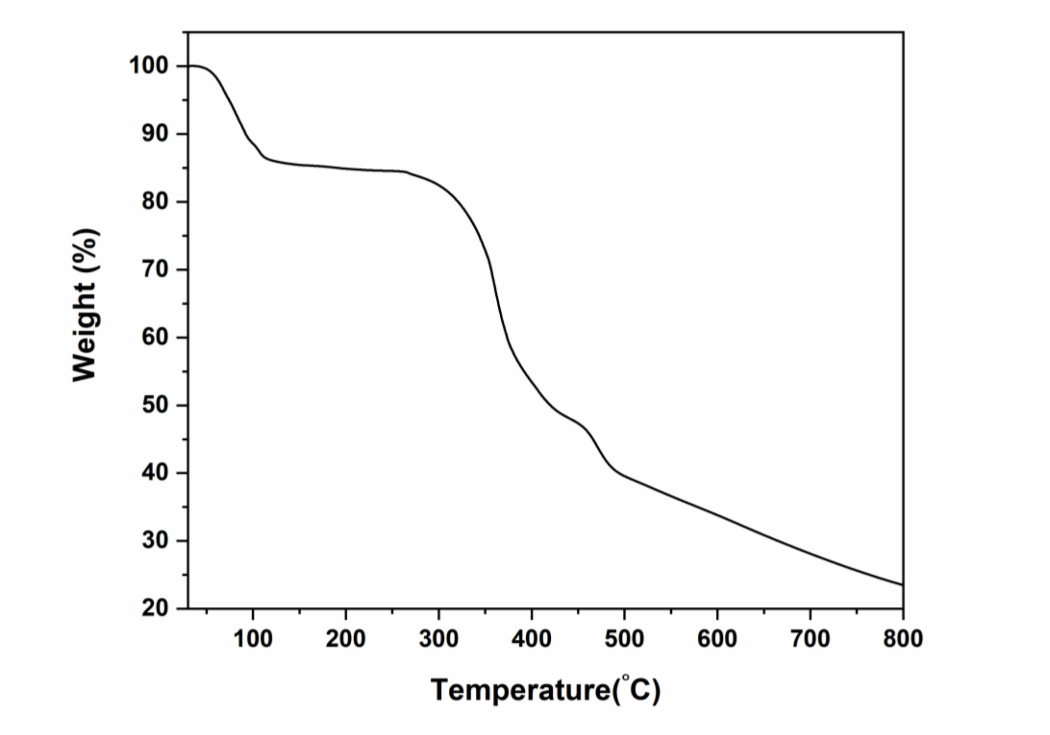

Fig. S12 TGA curve of the title compound.

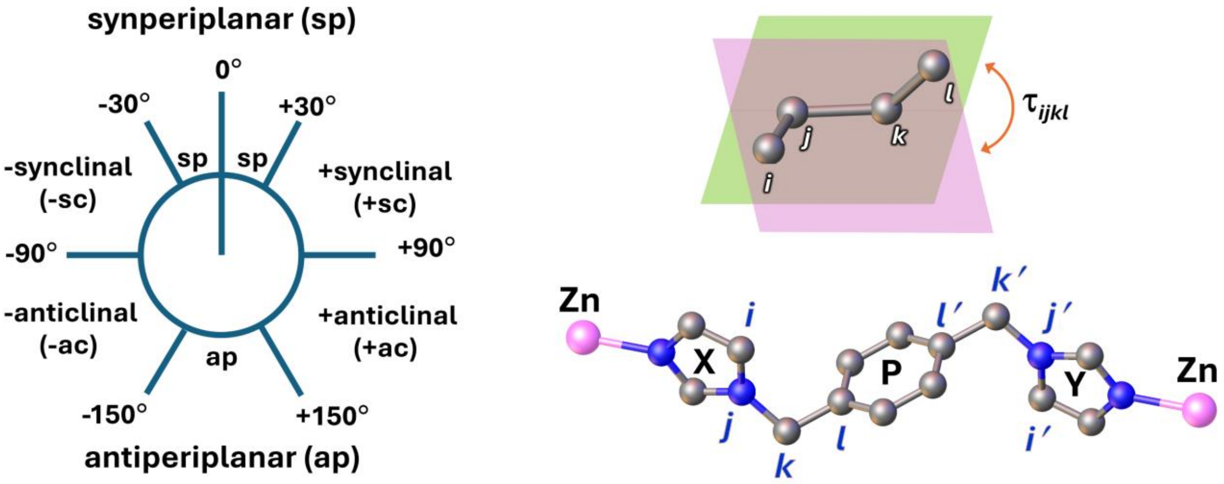

Scheme S1

Table S1 Geometrical parameters describing the conformations of bix ligands in the title compound (see also **Scheme S1**)

**bix ligand**	**Torsion angle, τ (°)**			**Angle between the aromatic rings (°)**			**Conformation**	**Zn···Zn distance (Å)**
	**τ_1*(ijkl)***	**τ_2 *(l′k′j′i′)***	**τ_3 *(jkk′j′)***	**X–Y**	**X–P**	**P–Y**		
{N2N3N4N5}	67.30	-83.80	7.90	119.17	58.75	67.21	*gauche*	11.262(1)
{N7N8N8N7}	-48.90	48.90	180.00	0.00	113.56	113.56	*trans*	15.099(3)
{N9N10N10N9}	78.70	78.70	160.10	30.94	73.02	73.02	*trans*	12.728(2)

Note: X and Y = imidazole rings and P = Phenyl ring
